# Face-valid phenotypes in a mouse model of the most common mutation in *EEF1A2*-related neurodevelopmental disorder

**DOI:** 10.1242/dmm.050501

**Published:** 2024-02-02

**Authors:** Grant F. Marshall, Melissa Fasol, Faith C. J. Davies, Matthew Le Seelleur, Alejandra Fernandez Alvarez, Cavan Bennett-Ness, Alfredo Gonzalez-Sulser, Catherine M. Abbott

**Affiliations:** ^1^ Centre for Genomic & Experimental Medicine, MRC Institute of Genetics and Molecular Medicine, University of Edinburgh, Western General Hospital, Crewe Road, Edinburgh EH4 2XU, UK; ^2^ Simons Initiative for the Developing Brain, University of Edinburgh, Edinburgh EH8 9XD, UK; ^3^ Centre for Discovery Brain Sciences, University of Edinburgh, Edinburgh EH8 9XD, UK

**Keywords:** Epilepsy, Translation, Autism, eEF1A2, Mouse

## Abstract

*De novo* heterozygous missense mutations in *EEF1A2*, encoding neuromuscular translation-elongation factor eEF1A2, are associated with developmental and epileptic encephalopathies. We used CRISPR/Cas9 to recapitulate the most common mutation, E122K, in mice. Although E122K heterozygotes were not observed to have convulsive seizures, they exhibited frequent electrographic seizures and EEG abnormalities, transient early motor deficits and growth defects. Both E122K homozygotes and *Eef1a2*-null mice developed progressive motor abnormalities, with E122K homozygotes reaching humane endpoints by P31. The null phenotype is driven by progressive spinal neurodegeneration; however, no signs of neurodegeneration were observed in E122K homozygotes. The E122K protein was relatively stable in neurons yet highly unstable in skeletal myocytes, suggesting that the E122K/E122K phenotype is instead driven by loss of function in muscle. Nevertheless, motor abnormalities emerged far earlier in E122K homozygotes than in nulls, suggesting a toxic gain of function and/or a possible dominant-negative effect. This mouse model represents the first animal model of an *EEF1A2* missense mutation with face-valid phenotypes and has provided mechanistic insights needed to inform rational treatment design.

## Teaser

**Summary:** A mouse model carrying the pathogenic E122K mutation in *Eef1a2* exhibits electrographic seizures and early motor abnormalities. Comparative phenotyping reveals a toxic gain-of-function or possible dominant-negative effect.

**Research Simplified**

Developmental and epileptic encephalopathies (DEEs) are rare disorders that usually arise in children. Patients with DEE can experience severe seizures and developmental delay, with treatments often not working. The cause of DEE in some patients is linked to mutations in a gene called *EEF1A2* that codes for the protein eEF1A2, which is important for the normal function of nerve and muscle cells.

The authors of this study introduced the genetic mutation most commonly found in patients into the *Eef1a2* gene in mice for the first time. Mice with mutated *Eef1a2* closely mirrored human DEEs, as they displayed problems with movement, decreased growth and, most importantly, signs of seizures, which have not been reported before in mouse models of the type of DEE associated with mutations in *EEF1A2*. This study expands the toolbox to study DEEs, which will enable further investigation of the cause of DEEs in patients with this *EEF1A2* mutation and could potentially lead to valuable new treatments.

## INTRODUCTION

Developmental and epileptic encephalopathies (DEEs) are rare neurodevelopmental disorders (NDDs) resulting from mutations in any one of over 200 genes (Helbig and Ellis, 2020), including *EEF1A2*. DEEs are characterised by early-onset seizures with developmental delay or regression. In addition to the debilitating seizure burden, they are associated with other challenges, including intellectual disability and behavioural problems, taking a substantial toll on those affected and their families (Gallop et al., 2021; Pressler and Lagae, 2020). Only a limited number of anti-seizure drugs are licensed for use in infants, and, although the rate varies by syndrome (McTague and Cross, 2013), many patients are refractory to treatment (Pressler and Lagae, 2020). There is a pressing need for new models of these disorders that show both construct and face validity in order to fulfil ambitions for precision medicine for severe, early-onset epilepsy (Knowles et al., 2022; Marshall et al., 2021).

*De novo* heterozygous missense mutations in *EEF1A2* resulting in DEEs and NDDs were first identified in 2012 using trio-based exome sequencing (de Ligt et al., 2012; Veeramah et al., 2013). Around 200 affected individuals have now been described in the literature or in clinical databases, with over 50 causative missense mutations found (Fig. S1). *De novo EEF1A2* mutations have now been estimated to occur with an annual incidence of 2.92 per 100,000 live births (López-Rivera et al., 2020), although the prevalence has not been estimated. The majority of cases have been identified in children, owing to growing use of whole-exome sequencing in paediatric populations presenting with early-onset epilepsies and neurodevelopmental deficits. However, given that genetic screening of adults with NDDs is not yet widespread (Zacher et al., 2021), a substantial cohort of undiagnosed adults with *EEF1A2* missense mutations is likely to exist.

Most individuals with heterozygous missense mutations in *EEF1A2* experience intractable seizures that often begin in the first year of life, are minimally or completely non-verbal, and have moderate to severe intellectual disability (Carvill et al., 2020; Lam et al., 2016; Nakajima et al., 2015). Diverse seizure types are seen, including infantile spasms, nodding spasms, typical and atypical absences, myoclonic seizures, and focal or generalised tonic-clonic seizures (Carvill et al., 2020; de Ligt et al., 2012; Lam et al., 2016; Wang et al., 2020). Microcephaly and structural brain abnormalities are also seen in some individuals, as are challenging behaviours and autism. Around half of the people with heterozygous mutations in *EEF1A2* are unable to walk and most of those with reduced mobility also present with movement disorders including ataxia, chorea and dystonia. Although several mutations have recurred in multiple cases, many have been found in only one individual (see Fig. S1). This makes genotype-phenotype comparisons difficult, but certain mutations are associated with more severe disease. Recent reports also suggest that some will develop a neurodegenerative course with poor long-term outcomes (Carvill et al., 2020). There is thus a real need for precisely targeted treatments in addition to standard anti-seizure medication.

The *EEF1A2* gene encodes the neuromuscular translation elongation factor eEF1A2. The canonical role of eEF1A proteins is the GTP-dependent delivery of aminoacylated tRNAs to the A-site of the ribosome during protein synthesis, functioning as part of the elongation factor 1 complex in conjunction with the GTP exchange factor complex eEF1B (Li et al., 2013). All vertebrates possess two independently encoded eEF1A isoforms (eEF1A1 and eEF1A2) which, despite being 92% identical at the amino acid level (Soares et al., 2009) and performing almost equivalently in translational assays (Kahns et al., 1998; Timchenko et al., 2013), have distinct spatiotemporal expression patterns. During early embryonic development, eEF1A1 is expressed ubiquitously and seemingly exclusively, with eEF1A2 later emerging alongside eEF1A1 in embryonic neurons and muscle (Davies et al., 2023). Postnatally, a conserved isoform switch occurs in which eEF1A2 completely replaces eEF1A1 in cardiac and skeletal muscle (Chambers et al., 1998; Lee et al., 1992, 1993; Pan et al., 2004) and partially replaces eEF1A1 in neurons; neuronal somata switch entirely to eEF1A2, with eEF1A1 thereafter restricted to axons (Davies et al., 2023). The eEF1A isoform switch is typically complete by around postnatal day (P) 21 in rodents (Chambers et al., 1998; Pan et al., 2004). The reason for this conserved tissue-specific switch between almost identical proteins remains unknown, but is hypothesised to relate to the differing non-canonical functional profiles of eEF1A1 and eEF1A2 (Mendoza et al., 2021; Mills and Gago, 2021).

A major consideration for *EEF1A2* missense mutations is to discover whether they broadly result in loss of function (LOF), gain of function (GOF), or both, as this would direct any therapeutic approach. Although over 50 causative missense mutations in *EEF1A2* have been described, no clear LOF variants (small deletions or frameshifts) have been reported, which is a highly unusual mutational profile suggestive of GOF (McLachlan et al., 2019). The missense mutations are scattered throughout the gene and are found in every coding exon (Fig. S1). Although there is some enrichment in the GTP hydrolysis and tRNA-binding domains, there is no strict clustering in particular domains from which we might infer the likely functional impacts. Disease models have revealed important functional insights about select *EEF1A2* mutations; however, the degree of LOF/GOF and the molecular mechanisms involved are only beginning to be established. For example, studies in yeast have concluded that the main mechanism for a number of variants is simple LOF (Carvill et al., 2020). Functional studies in mammalian systems are non-trivial, as all transformed, immortalised, induced pluripotent stem cell-derived, and primary cell cultures so far studied express eEF1A1, either exclusively or alongside eEF1A2, confounding genotype-phenotype correlations and potentially obscuring any LOF/GOF (McLachlan et al., 2019). A successful strategy we have previously employed is comparative phenotyping of mice carrying missense and null mutations in *Eef1a2* on the same genetic background (Davies et al., 2017, 2020). Comparison of weight gain profiles and neurological scores in mice homozygous for the D252H missense mutation and mice homozygous for null mutations revealed that mice carrying D252H were consistently more severely affected than nulls, suggesting a GOF. Mass spectrometry analysis revealed that D252H also prevents eEF1A2 from binding its cognate guanine exchange factor eEF1B, which is likely to cause a LOF (Davies et al., 2020). Although mouse models have thus revealed important functional insights, a key issue with those described so far is the lack of face-valid phenotypes (seizures, electroencephalographic abnormalities, motor abnormalities, learning and memory deficits, autism-relevant behaviours) with which to assess the efficacy of experimental therapies, particularly in the clinically relevant heterozygous missense mice. Mouse and human eEF1A2 only differ by a single amino acid, meaning that any of the causative missense mutations described so far can be straightforwardly modelled in mice, and recapitulating more clinically severe mutations may be more likely to produce models with face-valid phenotypes. The E122K mutation (c.364G>A, p.Glu122Lys) has been described in at least 11 individuals in the literature, making it the most commonly identified mutation. E122K presents with a consistently severe phenotype (see Table S1 for a summary of published clinical findings). All identified individuals with this mutation have early onset epilepsy, with onset typically in the first months of life and variable seizure control. There is strong evidence of epileptic encephalopathy in three cases, in which developmental arrest/regression occurred as the epilepsy progressed. All individuals have moderate to severe intellectual disability. Affected children typically have movement disorders, although many can walk. Most are non-verbal and several are reported to show self-injurious behaviour, sleep abnormalities and autistic behaviours.

We used CRISPR/Cas9 to generate a novel mouse model of the E122K mutation and carried out detailed phenotyping of both homozygotes and heterozygotes, alongside mice carrying null mutations on the same genetic background. Here, we describe early motor deficits, growth defects and electrographic seizures along with electroencephalogram (EEG) abnormalities in this model, and present evidence that E122K exerts a toxic GOF and/or possible dominant-negative effect.

## RESULTS

### Introducing the E122K mutation into the mouse *Eef1a2* gene

We used the clustered regularly interspaced short palindromic repeats (CRISPR) and CRISPR-associated 9 (Cas9) system to generate the E122K mouse line. A schematic of the CRISPR design for E122K missense incorporation is given in Fig. S2. Four F0 mice carried E122K alleles, complete with silent protospacer-adjacent motif (PAM) mutation and novel PstI site. Two of these F0 animals survived to breeding age and were mated with C57BL/6J stock, with one F0 animal transmitting the E122K allele to a single F1 female, which was used to establish the E122K breeding line. DNA from this F1 heterozygote was used to screen for off-target Cas9-induced mutations, with none detected. DNA from an E122K homozygote was later used for on-target analysis, confirming that there were no unintended edits to *Eef1a2* in exon 4 or its flanking introns (Fig. S3).

### The E122K mutation reduces protein levels in a tissue-dependent manner

**Fig. 1.**
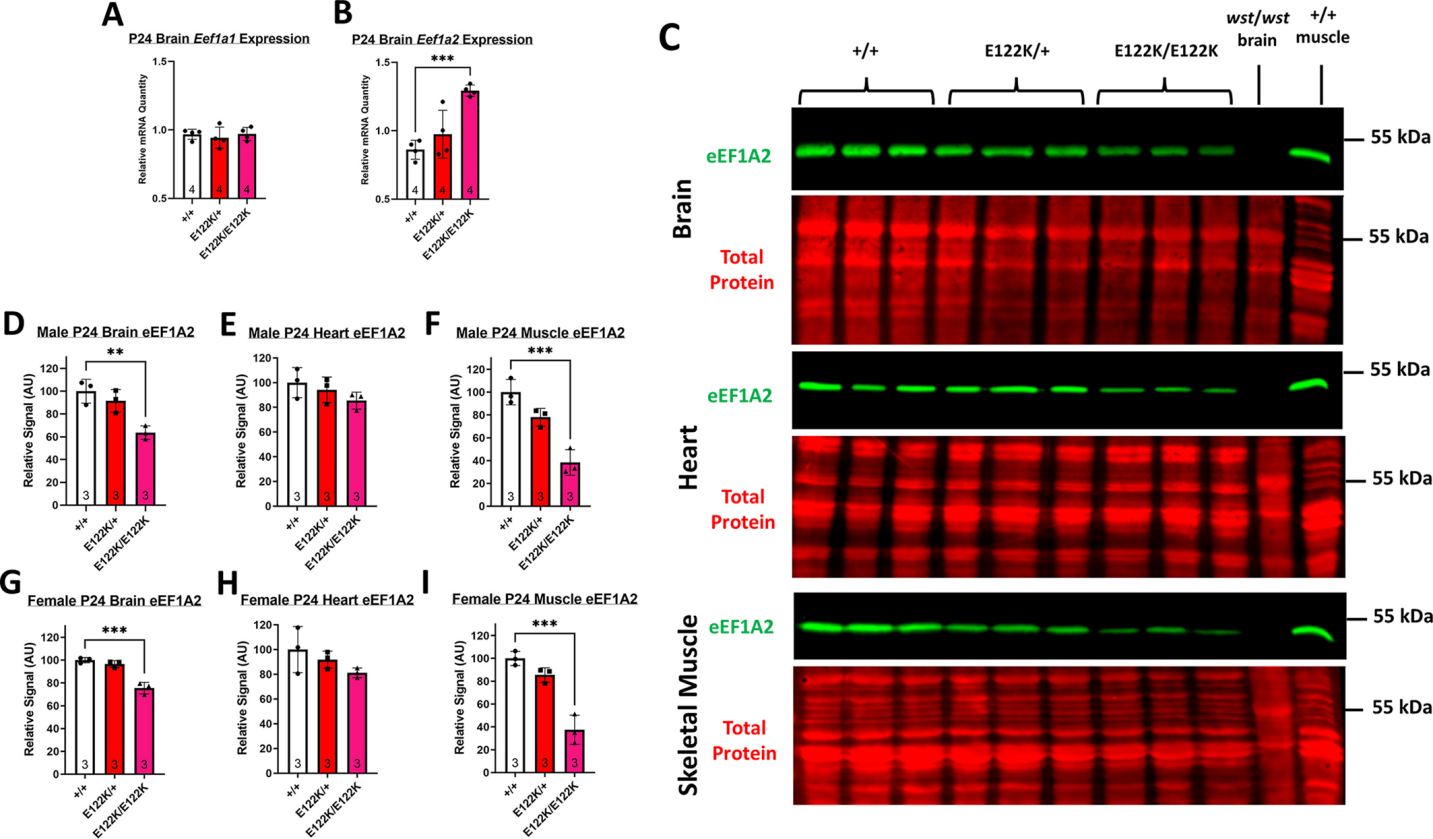
***Eef1a* expression analysis.** (A,B) Mean *Eef1a1* (A) and *Eef1a2* (B) mRNA quantities in the brains of P24 mice in the E122K line, normalised to the geometric mean of *Gapdh*, *Ubc* and *B2m* transcript levels. Values for individual biological replicates are shown as dots. Genotypes were compared using ordinary one-way ANOVAs. There was no statistically significant difference in relative *Eef1a1* mRNA quantities between genotypes [*F*(2, 9)=0.2807, *P*=0.7616]. There was a statistically significant difference in relative *Eef1a2* mRNA quantities between genotypes [*F*(2, 9)=16.16, *P*=0.001]. ***P<0.001 (Dunnett's post-hoc test, +/+ versus other genotypes). (C) Representative Western blots of P24 brain, heart and muscle lysates showing eEF1A2 and a total protein stain. Brain lysates from *wst/wst* mice, homozygous for an *Eef1a2* abolishing deletion (Chambers et al., 1998), were used as a negative control for eEF1A2. Lysates from adult skeletal muscle were used as a positive control. Brightness and contrast settings were set separately for each blot shown for illustration purposes (applied to the whole image), but this did not affect the quantification using Image Studio Lite software. Western blots were performed in triplicate for each sex with values averaged across replicates. (D-I) Quantification of eEF1A2 levels, normalised to total protein and expressed as a percentage of wild-type expression levels, were compared using ordinary one-way ANOVAs. There were no statistically significant differences in relative eEF1A2 levels in male heart [E; *F*(2, 6)=1.595, *P*=0.2782] or female heart [H; *F*(2, 6)=1.949, *P*=0.2227]. There were statistically significant differences in relative eEF1A2 levels in male brain [D; *F*(2, 6)=13.32, *P*=0.0062], female brain [G; *F*(2, 6)=39.60, *P*=0.0003], male muscle [F; *F*(2, 6)=28.49, *P*=0.0009] and female muscle [I; *F*(2, 6)=39.76, *P*=0.0003]. **P<0.01, ***P<0.001 (Dunnett's post-hoc test, +/+ versus other genotypes). Sample sizes are shown at the base of the bars. Error bars show the s.d. AU, arbitrary units.

All expression analysis was performed using tissue samples collected at P24, when the eEF1A isoform switch is typically complete in mice (Chambers et al., 1998; Pan et al., 2004). We found no difference in relative *Eef1a1* transcript levels in brains of mice with different genotypes (Fig. 1A), indicating that the E122K mutation causes neither a delay in the *Eef1a* isoform switch nor compensatory *Eef1a1* upregulation. Relative *Eef1a2* transcript levels were normal in E122K/+ mice but significantly increased in E122K/E122K mice (Fig. 1B). There was therefore no indication that E122K and the associated silent mutations reduce transcript stability, although there is upregulation of *Eef1a2* in homozygote brains.

We next estimated steady-state eEF1A2 protein levels in the brain, heart and skeletal muscle by western blotting (Fig. 1C). We found that the E122K mutation reduced mean steady-state eEF1A2 levels in all tissues, with significant reduction in E122K/E122K brains and skeletal muscle; however, these reductions were not uniform. Relative to wild type, eEF1A2 levels in E122K/E122K samples were on average 25-36% lower in the brain, 15-19% lower in the heart, and 62-63% lower in skeletal muscle, with intermediate levels in heterozygotes (Fig. 1D-I). The E122K mutation therefore reduces steady-state eEF1A2 levels in a tissue-dependent manner, with the greatest impact observed in skeletal myocytes. Further western blotting revealed no significant alteration in eEF1A1 levels in brain, heart or muscle of E122K/+ or E122K/E122K samples at P24, indicating no compensatory expression/upregulation of eEF1A1 (Fig. S4).

### Mice carrying the E122K mutation show growth abnormalities, with homozygotes dying by P31

Analysis of E122K/+ versus E122K/+ crosses revealed that males and females were born in the expected 1:1 ratio (χ^2^ test: χ^2^=0.25, *P*=0.61) and that +/+, E122K/+ and E122K/E122K mice were born in the expected Mendelian ratios in each sex (χ^2^ tests: male χ^2^=1.02, *P*=0.60; female χ^2^=3.37, *P*=0.19). To track early physical development, we measured total body mass of mice in the E122K line between P14 and P28. In order to gain mechanistic insight into the E122K mutation, we simultaneously characterised mice carrying *Eef1a2*-null mutations on the same genetic background (C57BL/6J). These mice were either heterozygous (+/−) or homozygous (−/−) for a 22 bp deletion in *Eef1a2* exon 3, which abolishes expression of eEF1A2 protein (Davies et al., 2020).

**Fig. 2.**
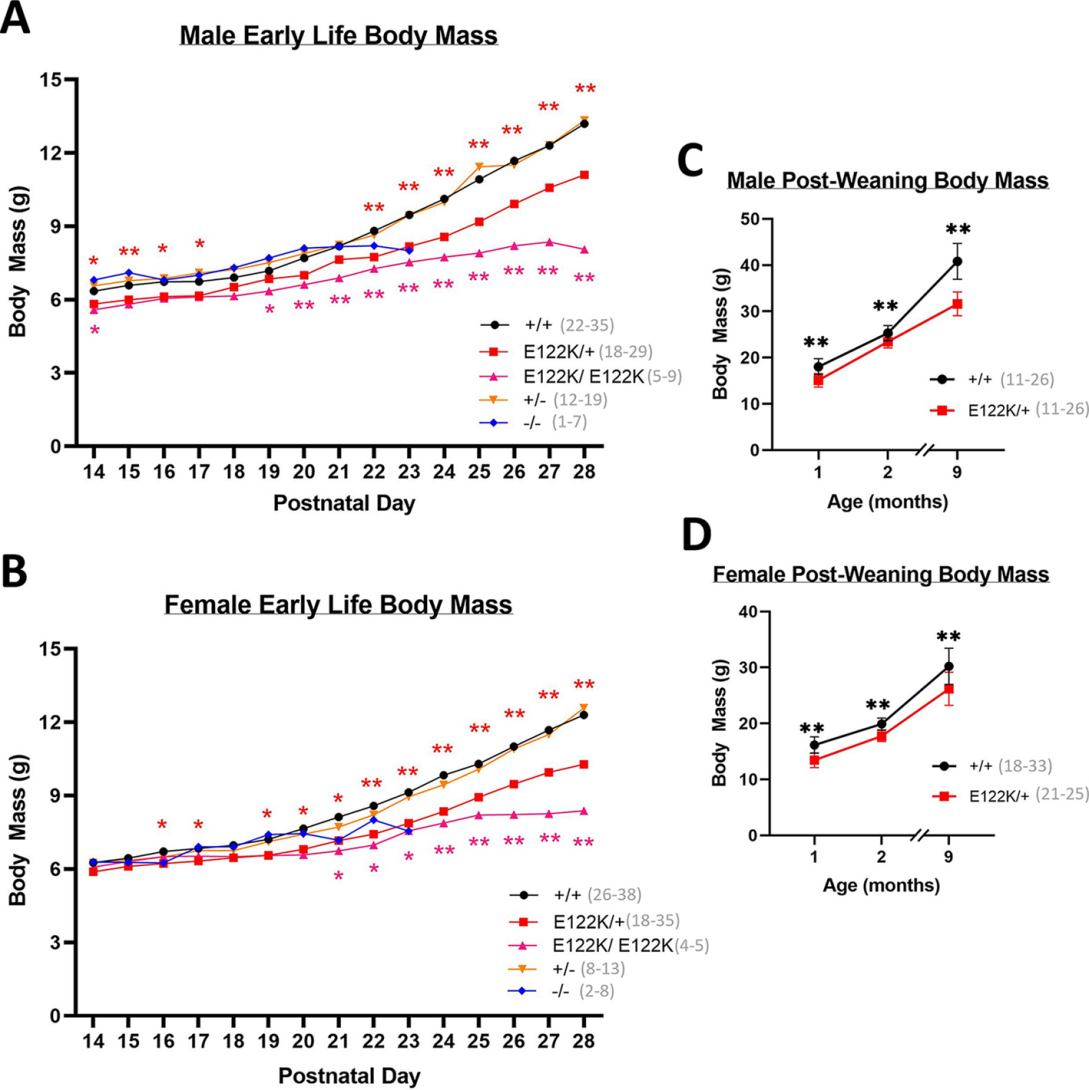
**Body mass deficits in mice carrying E122K.** (A,B) Mean absolute body mass in male (A) and female (B) mice between P14 and P28. (C,D) Absolute body mass in male (C) and female (D) mice between 1 and 9 months of age. At each age, genotypes were compared with wild type using Mann–Whitney *U*-tests followed by Bonferroni's correction for multiple comparisons. Statistically significant differences are denoted with colour-coded asterisks (*P<0.05, **P<0.01). Error bars in C and D show the s.d. Error bars have been omitted in A and B for illustration purposes. Sample sizes are indicated in panel keys. The entire experiment shown was performed once.

Although the gross morphology of E122K/+ and E122K/E122K mice was normal, we found that both E122K/+ and E122K/E122K mice developed body mass deficits in early life (Fig. 2). E122K/+ growth curves maintained an upwards trajectory but significantly deviated from those of +/+ mice from as early as P14 in males and P16 in females (Fig. 2A,B); by P28, E122K/+ body mass was on average ∼16% lower than that of wild types in both sexes. Statistically significant body mass deficits in E122K/E122K were detectable as early as P14 in males and P21 in females (Fig. 2A,B). E122K/E122K mice gained little weight after P21, and by P28 mean E122K/E122K body mass was ∼39% lower than that of +/+ in males and ∼32% lower than that of +/+ in females.

As previously reported (Davies et al., 2020), +/− mice show an essentially identical trajectory to +/+ mice, completely tolerating haploinsufficiency. −/− mice on the C57BL/6J background have stalled weight gain after ∼P20, exhibiting progressive motor/neurological abnormalities around P18 (Davies et al., 2020; Shultz et al., 1982) and reaching humane endpoints or dying of seizures by P23 (Davies et al., 2017, 2020). The timing of these −/− phenotypes is in line with the postnatal downregulation of eEF1A1 in mice, which is typically complete by P21 (Chambers et al., 1998; Davies et al., 2020; Khalyfa et al., 2001).

E122K/+ body mass deficits persisted throughout life, with mean E122K/+ body mass being ∼23% lower than that of +/+ mice in males and 13% lower than that of +/+ mice in females by 9 months of age (Fig. 2C,D). Adult E122K/+ mice of both sexes were noticeably leaner than their wild-type littermates, failing to develop the truncal adiposity typically seen in adult laboratory mice and remaining relatively lean even up to 18 months of age, even though mean daily food intake did not significantly differ between +/+ and E122K/+ mice at 2 months of age (Fig. S5). E122K/+ body mass deficits were also not driven by hypotrophy in eEF1A2-expressing tissues, as there was no significant decrease in the relative mass of E122K/+ brains, hearts or tibialis anterior muscles relative to body mass (Fig. S5). The mass of the kidneys (which do not express eEF1A2) was reduced in line with body mass in E122K/+ mice (Fig. S5), suggesting a consistent and proportional reduction in weight across all tissues.

Complete loss of eEF1A2 function in neurons causes vacuolar degeneration of spinal motor neurons in mice, beginning cervically and progressing rostrocaudally (Newbery et al., 2005) (Fig. S6C). Surprisingly, we did not observe the same in the cervical spinal cords of E122K/E122K mice at P28, which showed no clear degenerative pathological changes (Fig. S6A,B). These results indicate that the E122K protein retains sufficient function to spare motor neurons from overt degeneration until at least P28.

### Transient motor deficits in E122K/+ mice and progressive motor abnormalities in E122K/E122K mice

**Fig. 3.**
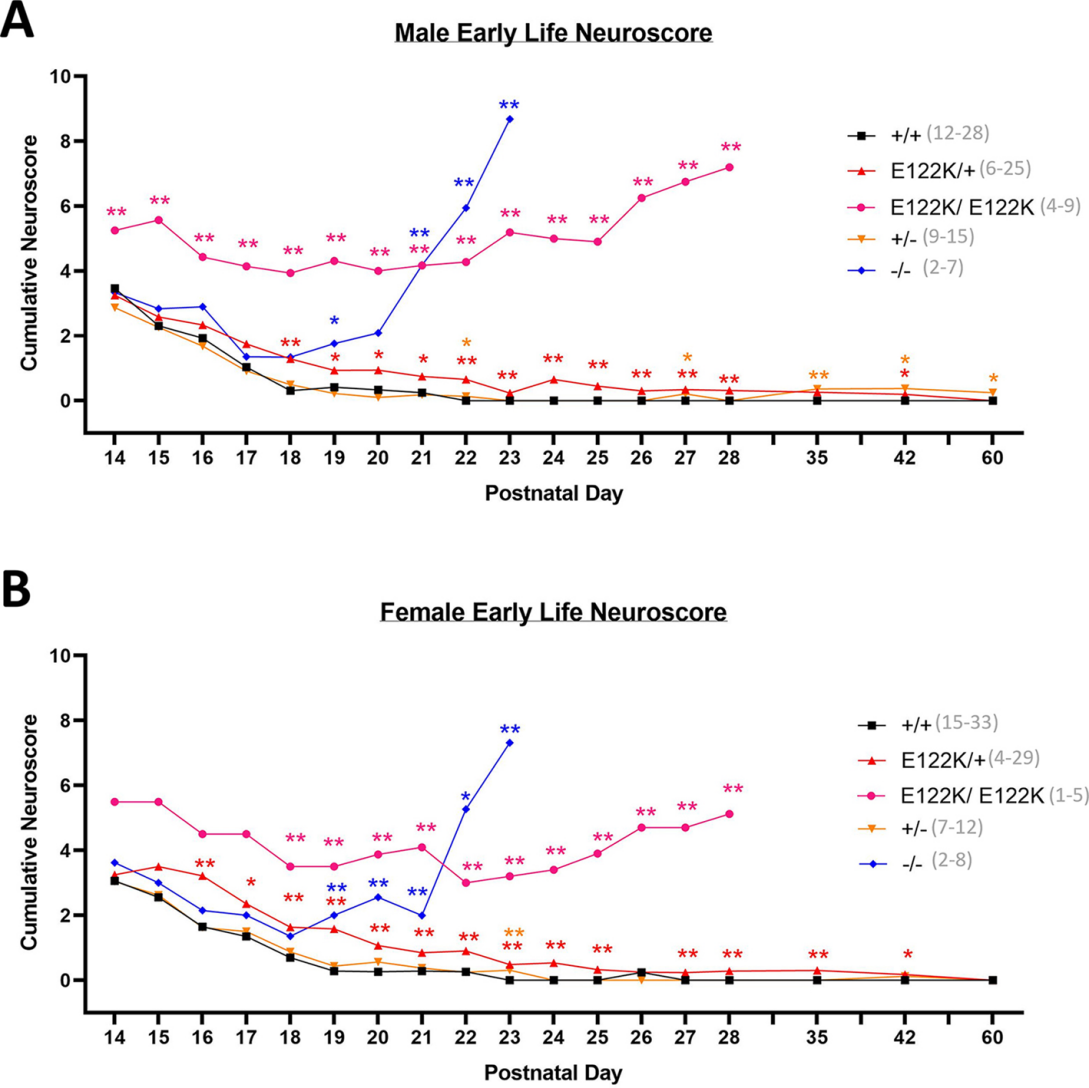
**Comparative neurological phenotyping reveals that E122K is toxic.** (A,B) Mean cumulative neuroscores in male (A) and female (B) mice measured between P14 and P60. At each age, genotypes were compared with wild type using Mann–Whitney *U*-tests followed by Bonferroni's correction for multiple comparisons. Statistically significant differences are denoted with colour-coded asterisks (*P<0.05, **P<0.01). Error bars have been omitted for illustration purposes. Sample sizes are indicated in panel legends. The entire experiment shown was performed once.

To track early motor/neurological development, we used a composite scoring system called the neuroscore, adapted from Guyenet et al. (2010) for scoring *Eef1a2* mutant mice (Davies et al., 2020). Cumulative neuroscores were measured between P14 and P60 (Fig. 3). In this scoring system, higher scores represent greater motor and neurological dysfunction. In each sex, +/+ mice began with neuroscores of ∼3, diminishing to ∼0 by 3 weeks of age as animals physically developed and learned the ledge walking task. The trajectory of +/− neuroscores was essentially indistinguishable from that of +/+ neuroscores, indicating that haploinsufficiency has no discernible impact on motor/neurological development. E122K/+ mice of each sex began with similar scores to those of +/+ mice at P14 but took longer to decline to a score of zero, remaining marginally but significantly elevated until at least P42 in males and females. The transient elevation of E122K/+ neuroscores was driven primarily by the gait and ledge walking components (Fig. S7), possibly representing a delay in motor development or changes in motor coordination as a result of the toxicity of the E122K protein.

Neuroscores in null homozygous mice were initially indistinguishable from those of +/+ mice but rose precipitously around 3 weeks of age, in line with the postnatal eEF1A isoform switch in mouse muscle (Chambers et al., 1998; Pan et al., 2004). As previously described, −/− mice develop progressive motor/neurological abnormalities between 2 and 3 weeks of age, reaching humane endpoints by P23 (Davies et al., 2017). Mean E122K/E122K neuroscores were significantly higher than those of all other genotypes at P14 in males and from P18 in females, remaining significantly higher than those of wild-type littermates thereafter (Fig. 3, data shown up to P28). The early E122K/E122K neuroscore was driven by gait abnormalities and difficulty walking along the ledge. Cervical kyphosis emerged around P18, with E122K/E122K mice exhibiting increasingly hunched/shuffling gaits and progressively losing their ability to walk along a ledge. In addition, E122K/E122K mice developed an essential tremor around P21. In spite of these abnormalities, E122K/E122K mice remained mobile and behaved normally in the cage, but were considered to be at humane endpoints when they progressed from stalled weight gain to weight loss, or began to exhibit reduced movement, declining body condition, grimace, or their tremor began to interfere with movement. E122K/E122K mice reached humane endpoints between P27 and P31.

**Fig. 4.**
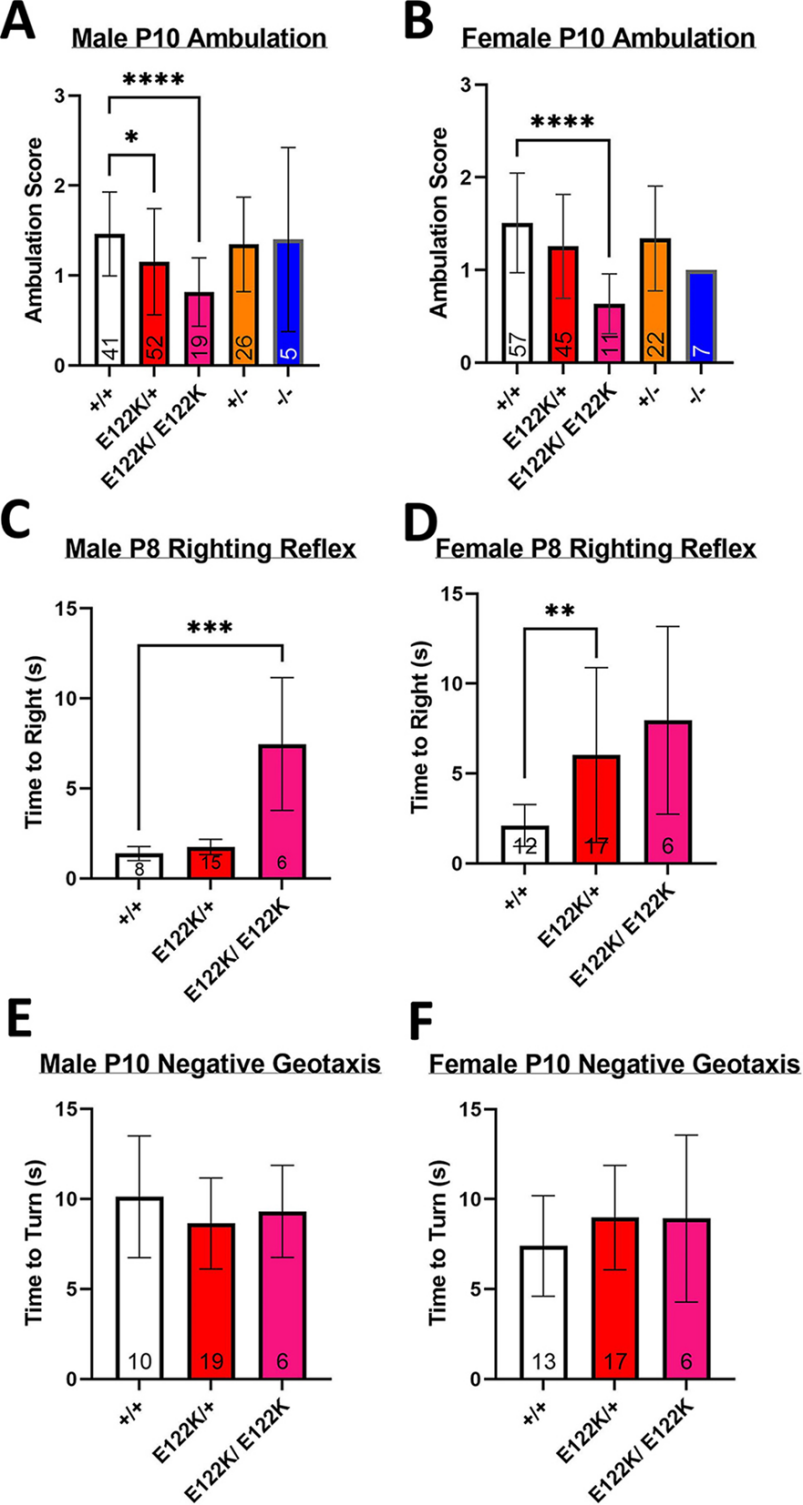
**Early motor deficits in neonates carrying E122K.** (A,B) Mean ambulation scores in male (A) and female (B) mice at P10. (C,D) Mean righting time for male (C) and female (D) mice at P8. (E,F) Mean turning time on the negative geotaxis apparatus for male (E) and female (F) mice at P10. Genotypes in A-C were compared using Kruskall–Wallis tests followed by Dunn's multiple comparisons test as appropriate (+/+ versus other genotypes, with +/− versus E122K/+ and −/− versus E122K/E122K in A,B). There was a statistically significant difference between genotypes in A (KW=21.7, *P*=0.0002), B (KW=25.57, *P*<0.0001) and C (KW=16.29, *P*=0.0003). Genotypes in D were compared using Welch's ANOVA, revealing a statistically significant difference between genotypes [*F*(2.000, 11.40)=4.789, *P*=0.0309], followed by Dunnett's T3 post-hoc test (+/+ versus other genotypes). Genotypes in E and F were compared using ordinary one-way ANOVAs, revealing no significant differences in males [*F*(2, 32)=0.9301, *P*=0.4049] or females [*F*(2, 33)=1.001, *P*=0.3784]. *P<0.05, **P<0.01, ***P<0.001, ****P<0.0001 in post-hoc tests. Sample sizes are shown at the base of the bars. Error bars show the s.d. The entire experiment shown was performed once.

Delayed motor development is a common feature amongst people heterozygous for E122K (Table S1). To investigate whether the E122K mutation was associated with motor abnormalities in mice, we performed a battery of neonatal motor tests consisting of ambulation scoring at P10, righting reflex tests at P8 and negative geotaxis tests at P10. Test protocols were adapted from Feather-Schussler and Fergusson (2016) (see Materials and Methods) and were performed by an experimenter unaware of genotype. First, ambulation was scored on an ordinal scale by observing P10 neonates as they explored an empty cage. Mean ambulation scores for +/+ neonates at P10 were ∼1.5 in both sexes (Fig. 4A,B), corresponding to a slow crawl with occasional symmetric limb movement. Ambulation scores in heterozygous and homozygous null mice did not significantly differ from those of wild-type mice, consistent with the fact that eEF1A1 remains highly expressed throughout the body at P10. However, ambulation scores were significantly lower in male E122K/+ neonates (mean score of 1.15) and in male and female E122K/E122K neonates (mean scores of 0.82 and 0.64, respectively). These E122K/+ and E122K/E122K scores correspond to (limited) crawling with only asymmetrical limb movement, consistent with a delay in motor development. We next tested the righting reflex in P8 neonates and found that P8 animals of all genotypes exhibited the righting reflex; however, male E122K/E122K pups and female E122K/+ pups took significantly longer than +/+ pups to flip back into the prone position (Fig. 4C,D). Wild-type neonates of each sex flipped to the prone position in <2.5 s on average, whereas female E122K/+ neonates took on average 6 s and male E122K/E122K neonates took on average 7.5 s. Although these results are not consistent with a delay in reflex development, they suggest that mice carrying the E122K mutation are delayed in developing the strength and/or coordination to flip from a supine to prone position, possibly in a sex-dependent manner. Lastly, we performed negative geotaxis tests in P10 neonates. All P10 mice tested were able to detect the incline and turn to face upwards, with no significant differences in the time taken to turn (Fig. 4E,F). In summary, this battery of neonatal motor tests revealed subtle and often sex-dependent early motor deficits that may represent delayed motor development in mice carrying the E122K mutation, with no evidence of vestibular deficits.

Hypotonia and progressive motor abnormalities have been found in humans carrying the E122K mutation (Table S1). To investigate whether mice show dystonic phenotypes, we measured grip strength (forelimb and all-limb) between P24 and 9 months of age but found no evidence of consistent grip strength deficits in E122K/+ or E122K/E122K mice (Fig. S8A-H). To investigate whether E122K/+ mice develop progressive motor abnormalities with age, we assessed their performance on an accelerating rotarod at 2 months of age (as a baseline) and at 9 months of age but found no evidence of declining rotarod performance over this age range (Fig. S8M-P).

### E122K/+ mice exhibit electrographic seizures and generalised spiking, but do not appear to have convulsive seizures

All individuals carrying the E122K mutation have epilepsy, with variable seizure types, ages of onset, and degrees of seizure control (Table S1). Other EEG abnormalities have been noted in several people carrying this mutation, including interictal spiking and spectral abnormalities. To investigate whether E122K/+ mice exhibit spontaneous seizures and/or EEG abnormalities, we performed intracranial EEG recordings in adult mice, with simultaneous electromyogram (EMG) recording and video capture.

Six E122K/+ mice (three male, three female) and six +/+ mice (three male, three female) were recorded. A total of 124 h 36 min of EEG data was collected between these 12 animals. The average amount of data collected did not significantly differ between sexes or genotypes (data not shown). Male and female data were combined for all analyses. There were no statistically significant differences between genotypes in the percentage of time spent in wake, rapid eye movement (REM) or non-REM (NREM) phases, and no statistically significant differences in NREM bout frequency, NREM bout duration or REM bout duration. However, there was a statistically significant 44% decrease in mean REM bout frequency in E122K/+ mice (Fig. S9), suggesting that E122K/+ mice might have altered REM sleep architecture.

**Fig. 5.**
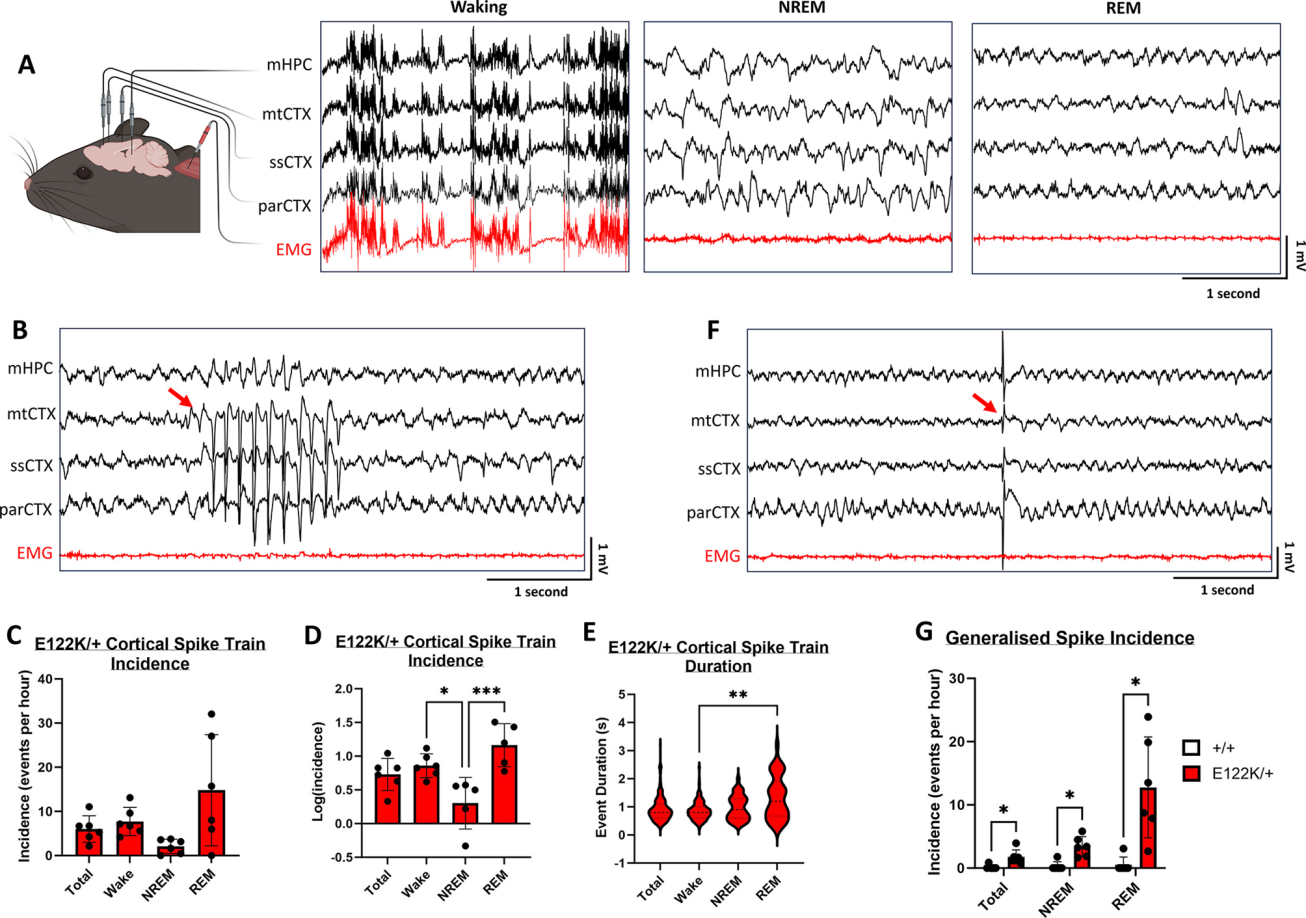
**Frequent electrographic abnormalities in adult E122K/+ mice.** (A) Representative polygraph traces showing EEG and EMG waveforms captured during waking, NREM sleep and REM sleep. EMG, electromyogram; mtCTX, motor cortex; mHPC, medial hippocampus; parCTX, parietal cortex; ssCTX, somatosensory cortex. (B) One 5-s epoch showing a CST occurring during REM sleep in an E122K/+ mouse. (C) Mean incidence (events per hour) of CSTs in total and across each across each vigilance state in E122K/+ mice (*n*=6). (D) Log transformation of the data shown in C to meet the assumptions of statistical tests. (E) Violin plots of CST duration in E122K/+ mice (*n*=6), in total and across each vigilance state. Dashed lines show the median and interquartile ranges. (F) One 5-s epoch showing a generalised spike occurring during REM sleep in an E122K/+ mouse. (G) Mean incidence (events per hour) of generalised spikes in total and across each across each vigilance state for +/+ (*n*=6) and E122K/+ (*n*=6) mice. Vigilance states in D were compared using one-way ANOVA, revealing a statistically significant difference between states [*F*(3, 18)=8.040, *P*=0.0013]. Vigilance states in E were compared using the Kruskall–Wallis test, revealing a statistically significant difference between states (KW=10.45, *P*=0.0151). Genotypes in G were compared within vigilance states using Welch's *t*-tests (two-tailed unpaired) followed by correction for multiple comparisons (Holm–Šídák method). *P<0.05, **P<0.01, ***P<0.001. Data in C were not statistically analysed. Error bars show the s.d. Mouse illustration in A was created with BioRender.com. Event onsets in B and F are marked by red arrows. The entire experiment shown was performed once.

No spontaneous convulsive seizures were observed during EEG recordings of E122K/+ mice, consistent with observations made during behavioural testing. Nevertheless, two putative EEG abnormalities (cortical spike trains and generalised spikes; see Fig. 5) were noted during vigilance state classification. The events here termed cortical spike trains (CSTs) were brief and relatively high-amplitude synchronous sharp polyspikes (∼7 Hz spiking frequency) in the motor and somatosensory areas, with occasional generalisation into the parietal cortex (Fig. 5B). CSTs were observed in all six E122K/+ mice recorded, with a total of 374 validated events and a mean incidence of approximately six events per hour [95% confidence interval (c.i.) 2.9-9.2 events per hour; Fig. 5C]. CSTs were observed in all vigilance states in E122K/+ mice, although there was significantly lower incidence in NREM sleep (Fig. 5C,D). CSTs had a median duration of 0.8 s [interquartile range (IQR 0.6-1.1; Fig. 5E], although REM CSTs had a significantly higher median duration than waking CSTs [1.2 s (IQR 0.98-1.9) during REM versus 0.8 s (IQR 0.6-1) during waking]. A total of three cortical polyspike events were observed between two +/+ mice (one male, one female), although these events did match the CST waveform (see Fig. S10), suggesting that CSTs are physiologically abnormal and possibly ictal events. Indeed, the CST waveform was reminiscent of the spike-wave discharges that are the hallmark of absence seizures (Papale et al., 2009; Pfammatter et al., 2019; Sadleir et al., 2006), yet also strikingly resembled the seizures observed in *Scn8a* mutant mice (Wagnon et al., 2015). CSTs had no apparent EMG correlates in either wake or sleep and were not consistently associated with altered EMG activity when they occurred during waking, although they often occurred during vigilance state transitions (see Fig. S10). To investigate whether CSTs had any behavioural correlates, the corresponding video was studied for a random selection of waking CSTs in E122K/+ mice (see Movie 1). We found that waking CSTs occurred during a range of normal behaviours, including exploration, rearing, digging, eating, grooming or other stationary activity, and were not associated with convulsions of any sort. The majority of waking CST events investigated coincided with rearing and sniffing, although not all bouts of rearing and sniffing coincided with CSTs. Although there appeared to be brief behavioural arrest during some events, behaviour generally continued uninterrupted. As CSTs were not consistently associated with Racine-scoreable behaviour, we conclude that CSTs are purely electrographic seizures.

The second EEG abnormality noted during vigilance state analysis was generalised spiking. Generalised spikes were single, high-amplitude spikes simultaneously affecting all EEG channels but not obviously driven by movement artefacts (Fig. 5F). The incidence of generalised spiking was significantly higher in E122K/+ mice (Fig. 5G), being observed in all six E122K/+ mice, with a total of 105 validated events and a mean incidence of 1.8 events per hour (95% c.i. 0.61-3.0 events per hour). A total of eight generalised spikes were observed in a single +/+ mouse (male) giving a mean incidence of 0.15 events per hour (95% c.i. −0.22-0.52 events per hour). In contrast to CSTs, generalised spikes were only observed during NREM and REM sleep (Fig. 5G). This may be physiologically relevant or simply reflect the fact that waking EEGs are relatively noisy, making it difficult to identify monospikes. Nevertheless, as these events were not identified during waking and did not have obvious EMG correlates, they were also presumed to be entirely electrographic.

Spectral abnormalities have been noted in several individuals with the E122K mutation. Notably, increased delta (1-4 Hz) activity in the parietal cortex during the waking interictal EEG has been concomitant with developmental arrest/regression in some people (Inui et al., 2016, 2020; see Table S1), representing a possible biomarker of encephalopathy. To investigate whether E122K/+ mice exhibit similar abnormalities, power spectra were generated for each vigilance state and EEG channel, with epochs containing CSTs and generalised spikes excluded. We found no statistically significant differences in power spectral density between genotypes in the delta (1-4 Hz), theta (5-10 Hz), sigma (11-16 Hz), beta (16-30 Hz), low gamma (30-48 Hz) or high gamma (52-100 Hz) spectral bands in any recording location or vigilance state (Figs S11 and S12).

### E122K/+ mice show normal object memory with possible anxiety signatures

To investigate the locomotor activity and anxiety-like behaviours in E122K/+ mice, we performed open-field tests at 1, 2 and 9 months of age. We found no statistically significant differences in total distance travelled between +/+ and E122K/+ mice of either sex between 1 and 9 months of age (Fig. S13A,B), indicating normal locomotor activity levels in out-of-cage contexts. There was no statistically significant difference in thigmotactic behaviour in males (Fig. S13C). However, female E122K/+ mice showed significantly increased thigmotactic behaviour at 1 and 9 months of age (Fig. S13D), covering 36% less distance in the centre zone than +/+ mice at 1 month and 45% less distance in the centre zone than +/+ mice at 9 months of age, suggesting increased anxiety levels in E122K/+ females (Gould et al., 2009).

All individuals with the E122K mutation exhibit moderate to severe intellectual disability (see Table S1). To investigate whether E122K/+ mice exhibited learning/memory deficits, we administered novel object recognition (NOR) tests at 1, 2 and 9 months of age and determined object discrimination indices for the sample and test trials based on the first 20 s worth of object interaction. We found no statistically significant differences in novel object discrimination indices in male or female E122K/+ mice between 1 and 9 months of age (Fig. S13K-N).

## DISCUSSION

Here, we used CRISPR/Cas9 to recapitulate precisely the clinical *EEF1A2* E122K mutation in mice and carried out detailed phenotyping of both homozygotes and heterozygotes, finding that mice carrying the E122K mutation exhibit early motor deficits, growth defects, and electrographic seizures alongside an increased rate of generalised interictal spiking. To unravel the pathogenesis of DEEs, disease models are required that can reveal the functional consequences of gene mutations. We used direct comparative phenotyping of mice carrying missense or null mutations in *Eef1a2* on the same genetic background, revealing that E122K does not simply phenocopy null mutations.

E122K/+ mice exhibit persistent body mass deficits and transient early motor deficits that are not seen in +/− mice, and E122K/E122K exhibit earlier onset body mass and neuroscore deficits than −/− mice, indicating that E122K exerts a toxic GOF and/or possibly a dominant-negative effect. Together with the fact that steady-state eEF1A2 levels were not significantly lower than those of +/+ mice in any E122K/+ tissues, the evidence is consistent with the view that the transient motor abnormalities and persistent body mass deficits in E122K/+ mice are driven by toxicity of the E122K protein and not by LOF.

E122K/E122K mice reached humane endpoints between P27 and P31, by which point they exhibited neuroscore phenotypes highly reminiscent of that seen in *Eef1a2* nulls. Progressive muscle wastage and motor abnormalities in *Eef1a2* null mice are associated with severe spinal cord pathology, which coincides with the downregulation of eEF1A1 (Newbery et al., 2005). Transgenic expression of eEF1A2 in null muscle is insufficient to rescue or even alter the trajectory of the degenerative phenotype (Doig et al., 2013), demonstrating that neuronal LOF is the main driver in the null context. Surprisingly, E122K/E122K mice showed no signs of spinal neurodegeneration by the time they reached humane endpoints and outlived nulls on the same genetic background by ∼1 week. This reveals that E122K is not a complete LOF mutation and, furthermore, it is not immediately clear what drives their progressive phenotype. Our western blots show that steady-state eEF1A2 levels are most reduced in E122K/E122K skeletal muscle, with estimated levels in E122K/E122K muscle being ∼60% lower than in +/+ muscle (versus only ∼30% lower than +/+ in neurons). Given this and the lack of obvious motor neuron pathology in E122K/E122K mice, we hypothesise that the primary driver of the progressive E122K/E122K phenotype is LOF in muscle, although it remains unclear if/how toxicity conferred by E122K contributes.

The tissue-specific impact of E122K on steady-state levels is intriguing and could be explained by several mechanisms that remain to be investigated, including reduced translation of the mutant mRNA, decreased stability of the E122K protein, and/or increased degradation of the E122K protein. Nevertheless, it is clear that decreased steady-state eEF1A2 levels in the brain are not secondary to mRNA instability, as relative *Eef1a2* mRNA quantities were unchanged in E122K/+ brains and significantly increased in the brains of E122K/E122K mice. This increase in homozygotes may represent compensatory upregulation in response to decreased eEF1A2 protein levels in homozygous neurons (El-Brolosy and Stainier, 2017).

The molecular bases of any LOF/GOF have yet to be fully established. E122K is adjacent to the GTP-binding site in the folded protein (see Fig. S1), meaning it is positioned to affect the kinetics of GTP binding or hydrolysis (Mita et al., 1997; Sandbaken and Culbertson, 1988). Recently, Mohamed and Klann (2023) investigated the effect of several pathogenic *EEF1A2* mutations, including E122K, on various eEF1A2 functions. Although they found no significant change in GTP hydrolytic activity of the E122K protein, they found that the presence of transiently transfected E122K protein significantly reduced *de novo* protein synthesis rates in HEK293 cells and primary mouse neurons, and significantly reduced translation elongation speed in HEK293 cells. They also found that E122K significantly increased binding to tRNAs, suggesting that the impact of E122K on translation could be the result of tRNA sequestration (Mohamed and Klann, 2023). Both increased and decreased translational speeds are associated with protein misfolding, with higher speeds being associated with higher rates of amino acid misincorporation (Wohlgemuth et al., 2010, 2011) and very low speeds associated with ribosome stalling and misfolding of nascent peptides (Ishimura et al., 2014; Komar and Jaenicke, 1995; Nedialkova and Leidel, 2015). Indeed, the E122K mutation was serendipitously characterised in the yeast *EEF1A* orthologue *TEF2* over 30 years ago, where it was shown to increase the rate of amino acid misincorporation (Sandbaken and Culbertson, 1988). Protein misfolding is associated with the accumulation of toxic aggregates (Powers and Balch, 2008), with neurons being particularly vulnerable (Knight et al., 2020). For example, *sticky* mice are homozygous for missense mutations in an alanyl tRNA synthetase causing mischarging of tRNAs, amino acid misincorporation, and protein misfolding, leading to rapid degeneration of cerebellar Purkinje neurons over the first months of life which results in severe tremors and ataxia (Lee et al., 2006). The E122K mutation also seems to have a neurodegenerative component, with progressive cerebral atrophy and ataxia noted in some individuals (see Table S1). Nevertheless, there were no obvious signs of neurodegeneration or progressive movement disorders in E122K/+ mice even up to 18 months of age.

The toxicity of E122K may also result from disruption of the non-canonical functions of eEF1A2. eEF1A2 has numerous non-canonical roles, including co-translational quality control of newly synthesised polypeptides (Gandin et al., 2013) and modulation of the actin cytoskeleton (Gross and Kinzy, 2005). With roles in protein synthesis and actin bundling, eEF1A2 appears to have an important role in synaptic plasticity, which depends upon both local protein synthesis in dendrites and local actin remodelling in response to different patterns of synaptic activity (Huber et al., 2000; Nakahata and Yasuda, 2018; Okamoto et al., 2004). Mendoza et al. recently showed that neuronal activity leads to the phosphorylation of eEF1A2, which causes dissociation from guanine-exchange factor eEF1B (attenuating local protein synthesis) and from F-actin (increasing actin dynamics), thereby coordinating the structural remodelling of dendritic spines with local protein synthesis rates (Mendoza et al., 2021). Interestingly, Mohamed and Klann (2023) found that E122K significantly decreased actin bundling *in vitro*, as well as significantly decreasing dendritic arborisation in transiently transfected mouse primary neurons, hypothesising that perturbed synaptic plasticity could underlie the neurodevelopmental syndrome.

One other intriguing possibility is that E122K results in toxicity by interfering with the function of isoform eEF1A1. As discussed, Mohamed and Klann (2023) showed that the E122K protein exerts toxic effects on HEK293 cells and primary mouse neurons, which were not observed when wild-type eEF1A2 was transfected in. HEK293 cells typically express eEF1A1 exclusively, whereas primary mouse neurons expressed both eEF1A isoforms even after several weeks in culture, meaning that the observed toxicity of the E122K protein could be explained as a pseudo-dominant negative against isoform eEF1A1. Consistent with this hypothesis, Mohamed and Klann (2023) found that the E122K protein had similar impacts on *de novo* protein synthesis and dendritic arborisation in mouse primary neurons isolated from both wild-type and *Eef1a2*-null mice. If a pseudo-dominant-negative effect against isoform eEF1A1 exists, the effect would be predicted to be most pronounced when both isoforms are highly expressed during early life. Interestingly, we observed that the E122K/+ neuroscore gradually declines to insignificance after P21 and the E122K/E122K neuroscore transiently ameliorates around P21, concomitant with the postnatal downregulation of eEF1A1 in mice, which is typically complete by P21 (Chambers et al., 1998; Khalyfa et al., 2001; Pan et al., 2004). Although this might simply reflect the impact of practice, it may also reflect decreasing toxicity as eEF1A1 is downregulated.

The E122K mouse model represents the first animal model of a clinical *EEF1A2* mutation to exhibit relevant, face-valid phenotypes that could be used to assess the efficacy of preclinical treatments. The electrographic seizures and increased interictal spiking observed in adult E122K/+ mice are directly relevant to the seizures and abnormal interictal spiking observed in human (see Table S1), and the neonatal motor deficits and transient neuroscore abnormalities observed in E122K/+ mice may be relevant to the delayed motor development and hypotonia observed in human. E122K/+ and E122K/E122K mice also showed robust and persistent growth defects. The E122K/+ mouse model will be valuable for preclinical proof-of-concept studies investigating different therapeutic approaches in *EEF1A2*-related neurodevelopmental disorder.

## MATERIALS AND METHODS

### Animal methods

All mouse work was carried out in accordance with the UK Animals (Scientific Procedures) Act 1986 using protocols approved by the local ethics committee of the University of Edinburgh. All mice (*Mus musculus*) used in this study were maintained on the C57BL/6JCrl genetic background and maintained as inbred colonies. All mice were housed in the Western General Hospital Biological Research Facility (BRF) except the mice used for EEG, which were housed in University of Edinburgh Centre for Discovery Brain Sciences facilities. Mice in the BRF were kept in Blue-Line 1285L IVC cages (Techniplast) with wood chips and tissue paper for bedding. Cages were enriched with a clear polycarbonate tube (Datesand) and a wooden chew stick. All mice were kept on a standard 12-h light 12-h dark cycle (light on at 07:00 h and off at 19:00 h) at 18-24°C and 45-65% humidity and had *ad libitum* access to food and water. Mouse pups were ear-notched between P12 and P14 for identification/genotyping and weaned after P21. Post-weaning animals were housed in single-sex mixed genotype cages containing two to five mice. Mice in the E122K line were typically supplied with mash diet at weaning to support the mutants.

All phenotyping and analysis were performed by male experimenters who were unaware of the genotype. Male and female animals were used in all experiments. Mice of each sex and genotype were generated in accordance with Mendelian inheritance. Behavioural and motor tests were performed on animals in single-sex, mixed genotype cages, with animals typically tested in the order of their colony number (randomly assigned at ear notching).

Throughout this article, mice heterozygous for the E122K mutation in *Eef1a2* (*Eef1a2*^+/E122K^) are referred to as E122K/+, and mice homozygous for the E122K mutation (*Eef1a2*^E122K/E122K^) are referred to as E122K/E122K. A second line of mice carrying the Del22Ex3 null mutation in *Eef1a2* (Davies et al., 2020) was studied alongside mice from the E122K line. Mice heterozygous for the Del22Ex3 mutation (*Eef1a2*^+/−^) are referred to as +/−, and mice homozygous for the Del22Ex3 null mutation in *Eef1a2* (*Eef1a2*^−/−^) are referred to as −/−.

### Generation of transgenic mice

The guide crRNA 5′-CGCCTCAAACTCGCCCACAC-3′ was selected by entering the genomic sequence of *Mus musculus Eef1a2* (assembly GRCm38.p6) into the CHOPCHOP tool (Labun et al., 2019). We designed two 200-nucleotide single-stranded oligodeoxynucleotide (ssODN) repair templates using the genomic sequence of *Eef1a2*. The first repair template contained three single nucleotide substitutions resulting in E122K missense, silently abolished the PAM to prevent repeated Cas9 mediated cleavage (Paquet et al., 2016), and silently incorporated a restriction site for the endonuclease PstI (facilitating convenient and low-cost genotyping of transgenic animals by PCR and subsequent restriction digest; Abbott, 1993). As biallelic mutations in *Eef1a2* cause postnatal lethality in mice (Chambers et al., 1998; Davies, 2017; Davies et al., 2017; Shultz et al., 1982), we designed and co-delivered a second repair template encoding only the silent PAM mutation in order to reduce the likelihood of generating such genotypes in the F0 generation (Davies et al., 2020). Repair templates were designed to be non-complementary to the crRNA (DiCarlo et al., 2013), and the introduction of rare codons (Zhao et al., 2021) was avoided.

The E122K ssODN repair template was: 5′-TCTTGTTGACACCCACAATGAGCTGCTTCACACCCAGAGTGTAGGCCAGGAGTGCGTGTTCCCGGGTTTGCCCGTTCTTGGAGATGCCCGCCTCAAACTTGCCCACACCTGCAGCCACGATCAGCACTGCGCAGTCCGCCTAGCCAACAGGTCAGACACAGTGAGTCCCCACCCGGCCCTGCCTTCGACCTGGCCCTGCC-3ʹ. The second ssODN repair template, with only silent PAM mutation, was: 5ʹ-TCTTGTTGACACCCACAATGAGCTGCTTCACACCCAGAGTGTAGGCCAGGAGTGCGTGTTCCCGGGTTTGCCCGTTCTTGGAGATGCCCGCCTCAAACTCGCCCACACCTGCTGCCACGATCAGCACTGCGCAGTCCGCCTAGCCAACAGGTCAGACACAGTGAGTCCCCACCCGGCCCTGCCTTCGACCTGGCCCTGCC-3ʹ.

E122K founder mice were generated by perinuclear microinjection of crRNAannealed totracrRNA (Integrated DNA Technologies, 20 ng/µl) and Cas9 nuclease (Integrated DNA Technologies, 1081058, 17 ng/µl) ribonucleoprotein complexes along with ssODN repair templates (Integrated DNA Technologies, each 75 ng/µl) into fertilised C57BL/6JCrl oocytes (Harms et al., 2014). Microinjections were performed by staff at the Evans Transgenic Unit (Western General Hospital, Edinburgh, UK). After 24 h, surviving embryos were implanted into pseudopregnant CD1-IGS female recipients.

### Genotyping

DNA was extracted from ear notches using the HotSHOT method (Truett et al., 2000). All Sanger sequencing was performed by staff at the IGMM Technical Services Sequencing Service using a 3130 or 3730 Genetic Analyser (Applied Biosystems). F0 animals were genotyped by amplifying exon 4 of *Eef1a2* with primers mE122KTOPOF and mE122KTOPOR and a proofreading polymerase (Platinum SuperFi DNA Polymerase, Invitrogen, 12351250), using the following thermocycler program: 98°C for 1 min, 35× (98°C for 5 s, 64°C for 10 s, 72°C for 15 s), 72°C for 2 min, 10°C hold. Blunt-end topoisomerase-based cloning was performed using the PCR products (Zero Blunt TOPO PCR Cloning Kit, Thermo Fisher Scientific, K2875J10), allowing the various *Eef1a2* alleles from each founder to be individually Sanger sequenced.

Further on-target analysis was performed by amplifying a 1025 bp region of *Eef1a2* from an E122K homozygote with primers mE122K1kbF and mE122K1kbR and a proofreading polymerase (Platinum SuperFi DNA Polymerase, Invitrogen, 12351250), using the following thermocycler program: 98°C for 1 min, 35× (98°C for 5 s, 54°C for 10 s, 72°C for 15 s), 72°C for 2 min, 10°C hold. PCR products were verified by electrophoresis then Sanger sequenced using the amplification primers.

To perform off-target analysis, the ten loci in the mouse genome with the highest probability of cleavage by *Streptococcus pyogenes* Cas9 using the selected guide crRNA were identified using CRISPOR (Concordet and Haeussler, 2018). PCR primers were designed for these ten loci (Table S2), which were amplified using a proofreading polymerase (Platinum Superfi II DNA Polymerase, Invitrogen, 12361010) using the following thermocycler program: 98°C for 1 min, 10× [98°C for 5 s, 65°C for 10 s (−0.5°C/cycle), 72°C for 15 s], 33× (98°C for 5 s, 60°C for 10 s, 72°C for 15 s), 72°C for 2 min, 10°C hold. PCR products were verified by gel electrophoresis then Sanger sequenced using the amplification primers.

Mice in the established line were genotyped by amplifying a region of *Eef1a2* centred on codon 122 with primers mE122KGenoF and mE122KGenoR and Taq DNA Polymerase (Life Technologies, 10342020), using the following thermocycler program: 95°C for 2 min, 10× [95°C for 15 s, 65°C (−1°C/cycle) for 20 s, 72°C for 50 s], 25× (95°C for 15 s, 55°C for 20 s, 72°C for 50 s), 72°C for 5 min, 10°C hold. PCR products were then digested using FastDigest PstI (Thermo Fisher Scientific, FD01614) for 2-5 min at 37°C then analysed using gel electrophoresis. Genotyping by PstI digest is illustrated in Fig. S2D.

### RNA expression analysis

Dissected tissues used for expression analysis were snap-frozen on dry ice then stored at −70°C until use. RNA was extracted from P24 brain tissue (frontal lobe) using the Direct-Zol Miniprep Plus kit (Zymo, R2070T) according to the manufacturer's instructions. Additional off-column DNase digestion was performed using the DNA-*free*™ DNA Removal Kit (Ambion, AM1906). RNA was converted to complementary DNA (cDNA) using the High-Capacity cDNA Reverse Transcription kit (Applied Biosystems, 4368814) according to the manufacturer's instructions. Primers for reverse-transcription PCR (RT-PCR) and quantitative RT-PCR (qRT-PCR) were supplied by Merck with standard desalting at 100 µM in TE buffer (Merck). Primers and cDNA samples were validated by RT-PCR with the primers given in Table S2 and Taq DNA Polymerase (Life Technologies, 10342020), using the following thermocycler program: 95°C for 2 min, 30× (95°C for 10 s, 60°C for 30 s, 72°C for 30 s), 72°C for 7 min, 10°C hold.

qRT-PCR reactions were performed using Brilliant II SYBR Green qPCR Master Mix (Agilent, 600828) according to manufacturer instructions using a Light Cycler HT7900 (Roche). For each qRT-PCR reaction, ∼2 ng of template cDNA was used, and the final concentration each primer was 300 nM. All reactions were run in triplicate, alongside −RT and water controls for each primer pair. In addition, standard curves were generated for each primer pair using a dilution series of wild-type cDNA (diluted 1:5, 1:50, 1:500, 1:5000 and 1:50,000). qRT-PCR reactions were run on the following lightcycler program: 50°C for 2 min, 95°C for 10 min, 40× (95°C for 30 s, 60°C for 1 min), 95°C for 15 s, 60°C for 15s.

qRT-PCR data were analysed using the HT7900 SDS software (v2.4.2). Absolute quantities of the target gene in each sample were extrapolated from the standard curves using the threshold cycle (Ct) values, and then normalised to the geometric mean of the reference genes *Gapdh*, *Ubc* and *B2m* (Vandesompele et al., 2002). Melt curves were generated for each primer pair at the end of the thermocycler program.

### Protein methods

For consistency, total protein was extracted from the parietal lobe of the brain, the whole heart, or a sample of hindlimb skeletal muscle containing biceps femoris, vastus lateralis and gastrocnemius muscle. Samples were lysed in a protein extraction solution (∼10 µl per mg tissue) consisting of 0.32 M sucrose with protease inhibitors (Merck, 4693159001) and phosphatase inhibitors (Pierce, A32957) using a Precellys-24 lyser. Lysed samples were centrifuged at 10,000 ***g*** for 15 min at 4°C and the supernatants collected. 2× Laemmli loading buffer was added in a 1:1 ratio then proteins were denatured by heating to 100°C for 5 min on a heat block and 10% (v/v) of 1 M dithiothreitol was then added to each sample. For SDS-PAGE, samples were electrophoresed through 10% acrylamide gels in Mini Gel Tanks (Invitrogen) filled with 1× tris-glycine-SDS running buffer, with 5 µl of Colour Prestained Protein Standard (New England Biolabs, P7719S) included on each gel. Wet transfers onto Immobilon-FL PVDF Membrane (Merck, IPFL00005) were then performed using 1× NuPAGE transfer (Invitrogen, NP00061) buffer in Mini Gel Tank Blot Modules (Invitrogen). Total protein was stained using REVERT 700 total protein stain (LI-COR, 926-11021) according to manufacturer instructions and visualised using a LI-COR Odyssey CLx Imaging System (169 µm resolution, low-quality setting). Blots were de-stained then blocked for 1 h at room temperature using INTERCEPT (TBS) Blocking Buffer (LI-COR, 926-11021). Blots were incubated in primary antibodies diluted in INTERCEPT (TBS) Blocking Buffer overnight at 4°C. Primary antibodies were a custom-made rabbit-anti eEF1A2 (1:2000; see below) or a custom-made sheep anti-eEF1A1 (1:1000) (Newbery et al., 2007). Blots were rinsed three times for 10 min each wash in TRIS-buffered saline+0.1% Tween 20 (TBS-T). Secondary antibodies were applied in INTERCEPT T20 Antibody Diluent (LI-COR, 927-65001) for 1 h at room temperature in the dark. Secondary antibodies were IRDye 680LT donkey anti-rabbit IgG (1:20,000; LI-COR, 926-68023) or IRDye 800CW donkey anti-goat IgG (1:20,000; LI-COR, 926-32214). After a second set of washes, blots were imaged as described above and were analysed using Image Studio Lite (version 5.2). Band intensities was measured against background and normalised to a standardised section of total protein within each lane. Uncropped blots are shown in Fig. S14.

### Generation of custom rabbit anti-eEF1A2 antibody

The custom rabbit anti-eEF1A2 primary antibody was manufactured to order by Proteintech (UK), and was equivalent to antibody eEF1A2-1 described by Newbery et al. (2007). The antibody was raised in a single rabbit by delivery of a peptide antigen (sequence KNVEKKSGGAGKVT), linked via a C-terminal cysteine to keyhole limpet haemocyanin carrier protein. Booster injections were performed after 28, 42, 60 and 78 days, with a final bleed 102 days after the first injection. Primary antibody was then affinity purified from host antiserum and frozen. The sequence KNVEKKSGGAGKVT corresponds to eEF1A2 residues 439-452.

### Spinal cord histology

Spinal cords were isolated and immersion-fixed in 4% paraformaldehyde (in PBS) overnight at 4°C then processed into paraffin blocks. Sections of 5 µm thickness were taken from the cervical regions using a Leica rotary microtome (RM2235) and mounted on glass microscope slides. Sections were stained with Haematoxylin and Eosin (H&E) using a standard protocol (Fischer et al., 2008) and coverslipped using a dibutylphthalate polystyrene xylene mounting medium. Brightfield images were acquired using a Hamamatsu C9600-02 NanoZoomer Digital Pathology microscope at 40× magnification using the default brightfield settings.

### Neuroscore assessments

The ‘neuroscore’ is a composite neurological function score originally designed for mouse models of spinocerebellar ataxia (Guyenet et al., 2010), but which has proven useful for phenotyping *Eef1a2* mutant mice (Davies et al., 2020). Briefly, hindlimb clasping, spinal kyphosis, ledge walking, and gait quality are individually scored between 0 and 3. These four scores are then summed to give a neuroscore ranging between 0 and 12, with higher scores representing more severe neurological phenotypes. The four neuroscore components were assessed as follows and in the following order, minimising the amount of tail handling required. All tests were conducted by an observer unaware of genotype.

#### Hindlimb clasping

The mouse was suspended by the base of the tail for 10-20 s and the position of the hindlimbs scored as follows: 0=hindlimbs point up and away from the abdomen with no clasping; 1=hindlimbs droop slightly or clasp slightly for more than half of the time suspended; 2=hindlimbs droop down and touch the abdomen than half of the time suspended; 3=hindlimbs are fully clasped and touch the abdomen for more than half the time suspended. Intermediate scores (e.g. 0.5 or 1.5) were given where appropriate.

#### Spinal kyphosis

The animal was next placed on the back of a gloved hand. The degree of spinal curvature was observed as the animal moved and sniffed and the spine was gently palpated. Animals were scored as follows: 0=no palpable curvature and able to fully straighten spine; 1=mild spinal curvature at rest but mostly able to straighten the spine; 2=pronounced spinal curvature at rest but only mild curvature when attempting to straighten spine; 3=pronounced spinal curvature that persists even when attempting to straighten the spine. Intermediate scores (e.g. 0.5 or 1.5) were given where appropriate.

#### Ledge test

The animal was lowered onto the ledge of an empty cage (Blue-Line 1285L IVC cage, Techniplast) with lid and food hopper removed. Mice were then allowed to walk along the edge of the ledge and then lower themselves into the cage. Both the ledge-walk and the landing contributed to the ledge score. If mice sat perched on the ledge without moving, they were encouraged with gentle nudging or by holding some used bedding tissue in front of them. Animals were scored as follows: 0=animal walks confidently along the cage edge and lands on its paws on the cage floor; 1=animal slips while walking, breaking its stride, but recovers without leaning off the ledge, or animal walks very reluctantly but without any obvious limb dragging (animal may land somewhat poorly, with its face or side making contact with the cage floor first); 2=animal moves stiffly, dragging hindlimbs along the cage edge, or slips while walking, leaning off the ledge, but recovering (animal may land very poorly, with its back making contact with the cage floor first); 3=animal cannot be encouraged to move along the ledge and eventually falls off, with a very poor landing. Intermediate scores (e.g. 0.5 or 1.5) were given where appropriate.

#### Gait assessment

After dropping from the ledge into the cage, mice were allowed to explore. A piece of tissue from the home cage was placed in the test cage to motivate locomotion. Gait was assessed as animals explored, with scoring as follows: 0=normal gait with body weight evenly distributed between all limbs, and hindlimbs pointing forward and held under the abdomen; 1=slight waddle or hunched posture with raised pelvis, with mild tremor; 2=waddling gait with hunched posture and raised pelvis, with hindlimbs pointing outwards and stuttering movements/tremor; 3=pronounced waddle with severely hunched posture and raised pelvis, with hindlimbs pointing outwards and pronounced tremor. Intermediate scores (e.g. 0.5 or 1.5) were given where appropriate.

### Neonatal motor tests

Testing procedures for ambulation scoring, righting reflex tests and negative geotaxis tests were adapted from (Feather-Schussler and Ferguson, 2016). In brief, for ambulation scoring, the degree of gait development of P10 pups was scored on an ordinal scale between 0 and 3, with higher scores representing more developed gaits. For righting reflex tests, P8 pups were placed in a supine position and the time to return to a prone position was measured. For negative geotaxis tests, P10 pups were placed facing down a 45° incline and the time taken to turn and face up was measured. Righting reflex and negative geotaxis measurements were performed three times per pup with animals returned to the home cage for a minimum 2-min break between tests.

### Grip strength tests

Grip strength was measured using an electronic grip strength meter (Bioseb) as previously described (Mandillo et al., 2008). The grip strength of the forelimbs was measured three times back-to-back then the mean was calculated. After a 2-min break, all-limb grip strength was measured three times back-to-back, then the mean was calculated.

### Accelerating rotarod tests

Motor function and coordination was tested using an accelerating rotarod (Bioseb). The rotarod beams were covered with a soft low-pile carpet material for grip. The beam was accelerated from 4 to 40 rpm over 2 min. Mice were given three rotarod trials per day over four consecutive days, with a 15-min break between trials. Maximum trial duration was 5 min. Mice failed the rotarod trial when they either slid off the beam or spun freely with the beam for a total of two rotations, which did not have to be consecutive. The mean latency to fail was determined on each day for each animal.

### Open-field tests

Open-field tests were performed in a white Perspex box with dimensions (width×depth×height) of 50×50×40 cm. The arena was evenly illuminated (∼226 lux) by two shaded 3600 K 40 W light bulbs suspended 90 cm above the box floor. A Logitech C270 HD webcam was mounted 90 cm directly above the centre of the arena to record the sessions (960 p resolution, 30 frames per second). Open-field tests were performed at 1, 2 and 9 months of age. All animals at were naïve to the arena at 1 and 2 months, whereas the 9-month-old cohort consisted of a mixture of naïve and non-naïve animals. Recordings were batch-analysed using the open-source EzTrack video analysis software (Pennington et al., 2019). First, a 30×30 cm region of interest in the centre of the box was digitally annotated for thigmotaxis measurements. Next, animal tracking was performed using the following analysis parameters: Loc_thresh=99; Use-window=True; Window_size=100; Window_weight=0.5; Bin_dict=none. Distance travelled (pixels per frame) and distance travelled in the centre zone (pixels per frame) were extracted from the EzTrack output.

### NOR tests

NOR tests were performed at 1, 2 and 9 months of age in the same arena as open-field tests, following three consecutive days of brief exposure to the arena to familiarise the animals (Leger et al., 2013). NOR tests took place on the fourth day, on which mice were allowed to acclimatise to the testing room for at least 30 min. Mice were always tube-handled into and out of the arena, and the arena and objects were wiped down with 70% ethanol between animals. NOR trials were performed and scored by one of two experimenters (unaware of genotypes), who trained together to score object interaction (sniffing, climbing, chewing, or rearing against the objects). Inter-rater reliability was assessed by repeat scoring of a set of ten trial recordings. A sample trial (with two identical objects) took place first, followed by a test trial (with one familiar object and one novel object) after a 4-h intertrial interval. The object acting as the novel object, as well as the side that the novel object was presented on, were randomly assigned to animals. Sample and test trials were scored as described by Leger et al. (2013). In both the sample and test trials, the amount of time animals spent interacting with each object was timed using stopwatches (rounded to the nearest second) until a total of 20 s of object interaction was reached. The latency to reach 20 s of interaction was recorded for each animal. If animals failed to meet 20 s of object interaction within 10 min they were excluded. For each trial, a discrimination index (DI) was then calculated based on the first 20 s of object interaction [DI=(difference in object interaction time)/20]. For the test trial, a DI was calculated for the novel object such that exclusive interaction with the novel object would give a DI of 1. For the sample trial, a DI was calculated for the left-hand side object. Animals with DI≥0.3 in the sample trial were excluded as they were deemed to be showing place preference. Mice tested at 1 and 9 months of age were all naïve to the test/objects. Data at 2 months of age was collected from a mixture of naïve and non-naïve mice. As object recognition memory in mice decays to chance within days (Bolz et al., 2015; Leger et al., 2013; Moore et al., 2013), the same objects were used for tests of non-naïve mice, but as a precaution the object used as the novel object was switched.

### EEG/EMG electrode implantation surgery

Electrode implantation surgeries were performed after 6.5 weeks of age. Animals were anaesthetised with isoflurane and mounted on a stereotaxic frame (David Kopf Instruments, USA). Pairs of local field potential (LFP) electrodes (Teflon coated stainless steel, diameter=50.8 μm; A-M Systems, USA), support screws and electrical ground screws (Yahata Neji, M1 Pan Head Stainless Steel Cross, RS Components, Northants, UK) were implanted at the coordinate locations given in Table 1.

**Table 1.**
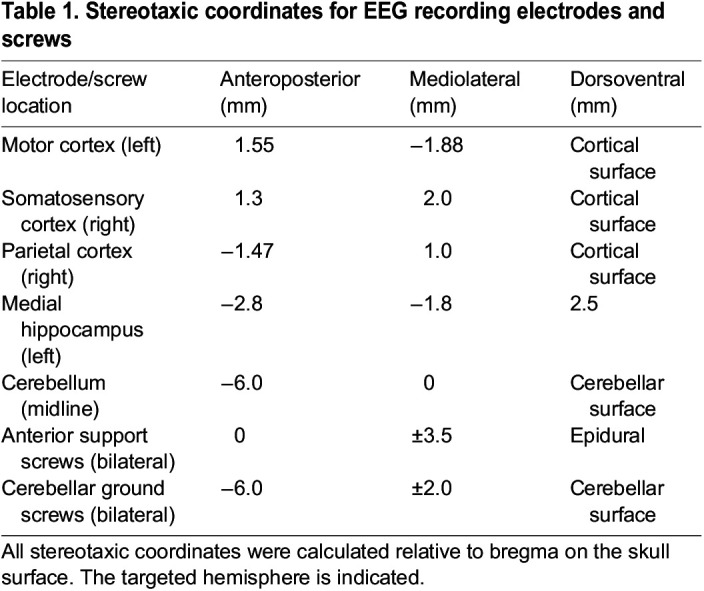
Stereotaxic coordinates for EEG recording electrodes and screws

Implanted electrodes were sealed in place using ultraviolet light-activated dental cement (3 M Relyx Unicem 2 Automix, Henry Schein, UK). The electrodes were pinned to an electrode interface board (EIB-16. Neuralynx, USA) which was mounted on the top of the skull using dental cement. Finally, a stainless-steel wire was sutured onto the trapezius muscle for EMG recording. Animals were allowed to recover for at least 7 days before tethered recordings took place. Analgesia (Carprofen) was given peri-operatively and then post-operatively as necessary.

### Tethered polygraph and video recordings

Tethered EEG recordings took place between 7.5 and 12.5 weeks of age. Animals were continuously recorded during the light phase for 3.5-6 h on at least two different days. Recordings took place inside (width×depth×height) 50×50×40 cm Perspex arenas, with wood chips as bedding. A small pot of water and some chow pellets were added to each arena. Up to four animals were recorded at once, with the animals unable to see one another and the experimenter unaware of genotype. The electrode interface boards were tethered to RHD 16-channel recording headstages (Intantech, USA) wired to an acquisition board (OpenEphys, USA) via an electrical commutator (Adafruit, Italy). LFPs were recorded in the OpenEphys continuous format with a sampling frequency of 1 kHz (high pass filter >7500 Hz and low-pass filter <2 Hz) and referenced to ground. Mice were simultaneously video recorded from above at ∼10 frames per second using a C270 HD Webcam (Logitech, USA).

### Analysis of EEG/EMG recordings

EEG/EMG analysis was performed in Python (V2.6.6) using custom code based on the MNE-Python package (V1.0.0) (Gramfort et al., 2013). First, continuous EEG/EMG recordings were split into 5 s epochs. An experimenter unaware of genotype classified epochs as wake, NREM or REM using the EEG and EMG waveforms using the following criteria based on EEG/EMG characteristics. Wake was identified by the presence of desynchronised EEG and varying levels of EMG. NREM epochs displayed high-amplitude slow-wave (∼1–4 Hz) EEG activity accompanied by sleep spindles (∼12–17 Hz) and decreased EMG activity. REM was identified by sustained theta (∼5–10 Hz) and no EMG activity. The percentage of time in wake, NREM and REM, as well as the frequency of NREM/REM bouts and the latency to REM were then calculated for each animal. NREM bouts were defined as any transition from waking or REM to NREM. REM bouts were defined as any transition from NREM to REM. Epochs with putative abnormalities (monospikes or polyspikes) were noted during vigilance state classification and were later revisited by an experimenter unaware of genotype for validation as normal, abnormal or artefactual. Precise onset and offset times for validated polyspikes were determined and the mean duration was quantified for each animal. For video analysis of CSTs, polygraph and video recordings were synchronised using file metadata. Alignment was verified for each recording by studying sleep-to-wake transitions.

### Spectral analysis of background EEG

EEG data from each animal was sorted by recording location and vigilance state. Epochs were Hanning-tapered and then Fourier-transformed using Welch's Fast Fourier Transform (Welch, 1967) using SciPy (Oliphant, 2007) with a 50% window overlap to obtain the power spectral density (PSD) at each frequency between 1 and 100 Hz. Epochs with CSTs or generalised spikes were excluded. Epochs containing visually identified artefacts (movement or electrical interference) or containing mixed/transitional vigilance states were also excluded from spectral analysis. For statistical comparisons, average PSDs for each animal were calculated for the delta (1-4 Hz), theta (5-10 Hz), sigma (11-16 Hz), beta (16-30 Hz), low gamma (30-48 Hz) and high gamma (52-100 Hz) spectral bands.

### Statistical analysis

Statistical analyses were performed in GraphPad Prism (Version 9), SPSS (Version 25) and R (Version 4.0.5). For all data that were statistically analysed, outlier removal was performed using the ROUT method (Motulsky and Brown, 2006), with a *Q* value of 1%. The normality of residuals was tested using the D'Agostino–Pearson omnibus (K2) test or Shapiro–Wilk test, as appropriate. The equality of variances/homoscedasticity of data was also tested using Bartlett's test of homogeneity of variances, the Brown–Forsythe test or Spearman's rank correlation test, as appropriate. Appropriate parametric or non-parametric statistical tests were then performed. Parametric tests included one- and two-way ANOVA and two-tailed unpaired *t*-test (with or without Welch's correction). Non-parametric tests included the Mann–Whitney *U*, Kruskall–Wallis and χ^2^ tests. Post-hoc testing [Dunn's multiple comparisons test or Dunnett's (T3) post-hoc test] and correction for multiple comparisons (Bonferroni's correction or Holm–Šídák method) were performed as appropriate. Data transformation was performed where necessary to meet the assumptions of statistical tests. Individual animals were the statistical unit for all tests unless otherwise stated.

## Supplementary Material

10.1242/dmm.050501_sup1 Supplementary information
