# Supplementary material for: Face-valid phenotypes in a mouse model of the most common mutation in EEF1A2-related neurodevelopmental disorder
Source: Dis Model Mech. 2024 Feb 2;17(6):dmm050501. doi: 10.1242/dmm.050501 (PMC10855229; doi:10.1242/dmm.050501)
Supplement: Supplementary information [file dmm-17-050501-s1.pdf]

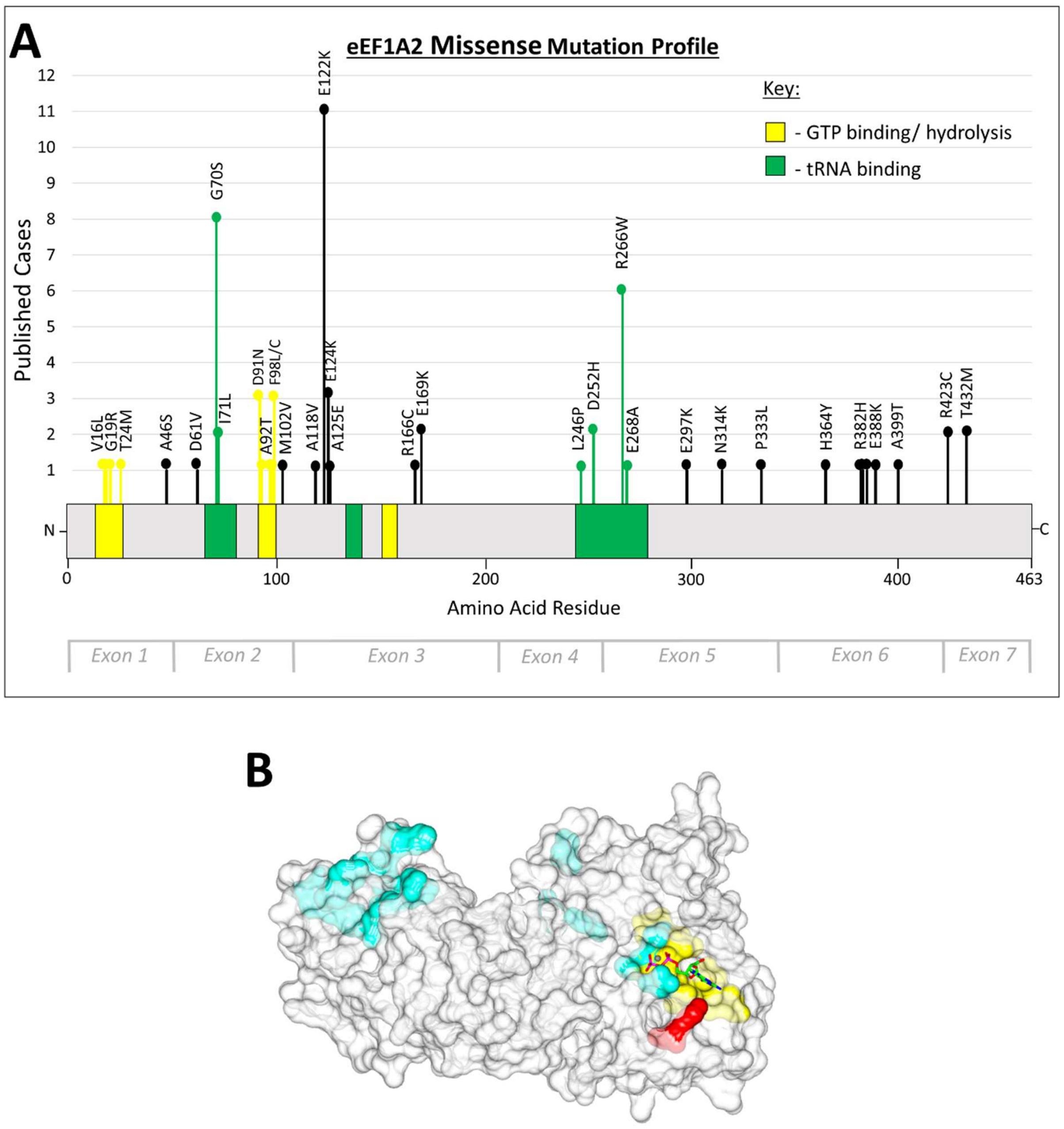

Fig. S1. Mutational profile of *EEF1A2* and location of E122K on 3D structure. A: mutational profile of *EEF1A2* (published cases as of end 2021). **B**: 3-dimensional structure of mammalian eEF1A2 protein (PDB:4C0S) in the GTP-bound conformation, showing codon 122 (red), the GTP binding site (yellow) and the eEF1B binding site (cyan). Rendered in CCP4.

Disease Models & Mechanisms • Supplementary information

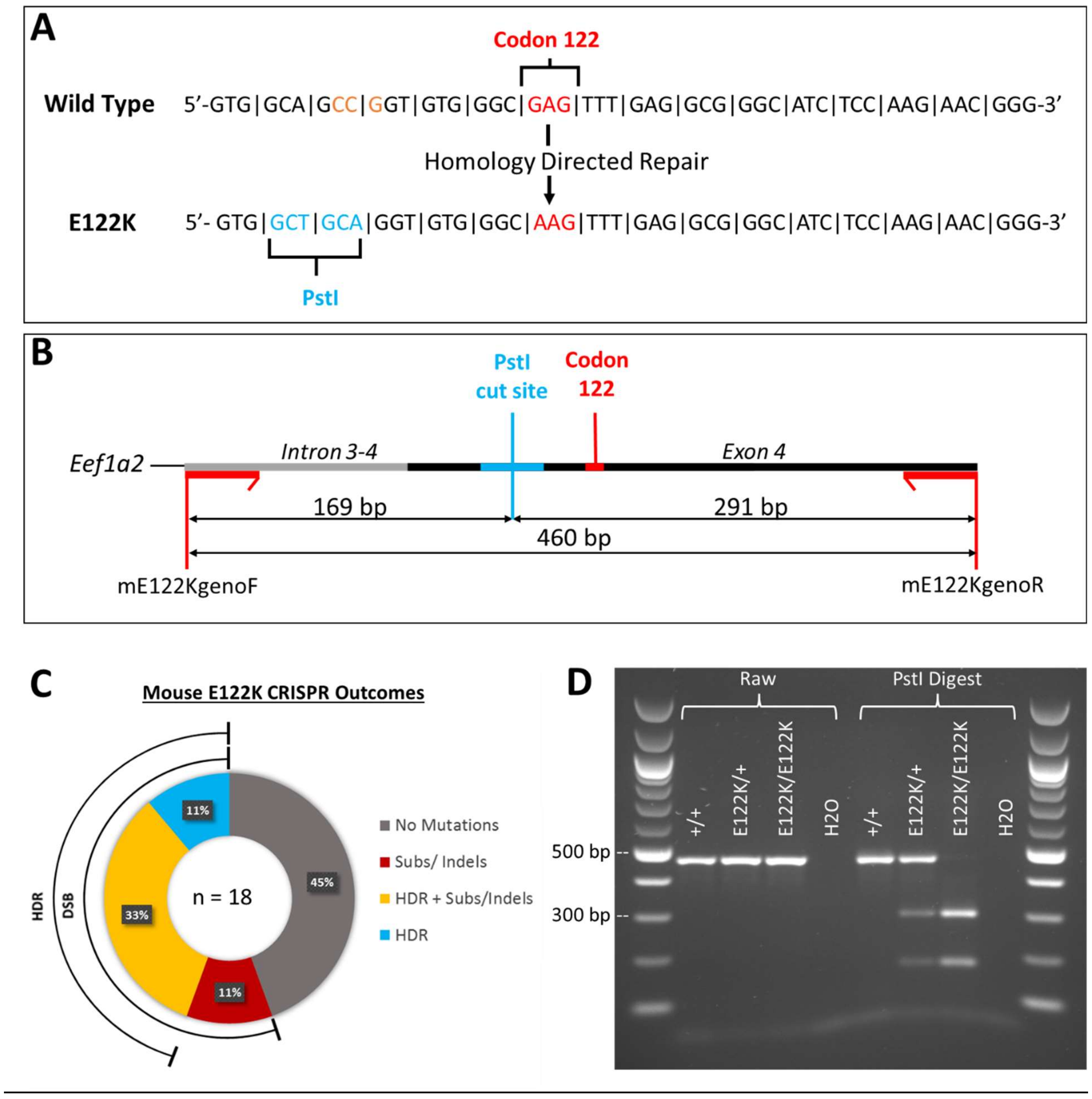

Fig. S2. design and outcomes of E122K CRISPR experiment. A: Schematic of DNA changes made to *Eef1a2*, showing codon 122 (red) and the silently incorporated PstI site (cyan). The nucleotides complementary to the PAM are highlighted in orange in the wildtype sequence. **B**: Schematic of the region of genomic *Eef1a2* amplified by primers mE122KgenoF and mE122KgenoR, for genotyping mice in the established line. **C**: breakdown of founder mice by CRISPR outcomes. HDR = homology directed repair. DSB = double stranded break. **D**: Agarose gel showing (left) raw PCR products amplified from +/+, E122K/+ and E122K/E122K ear notch DNA and (right) the same PCR products following restriction digest with PstI.

Disease Models & Mechanisms • Supplementary information

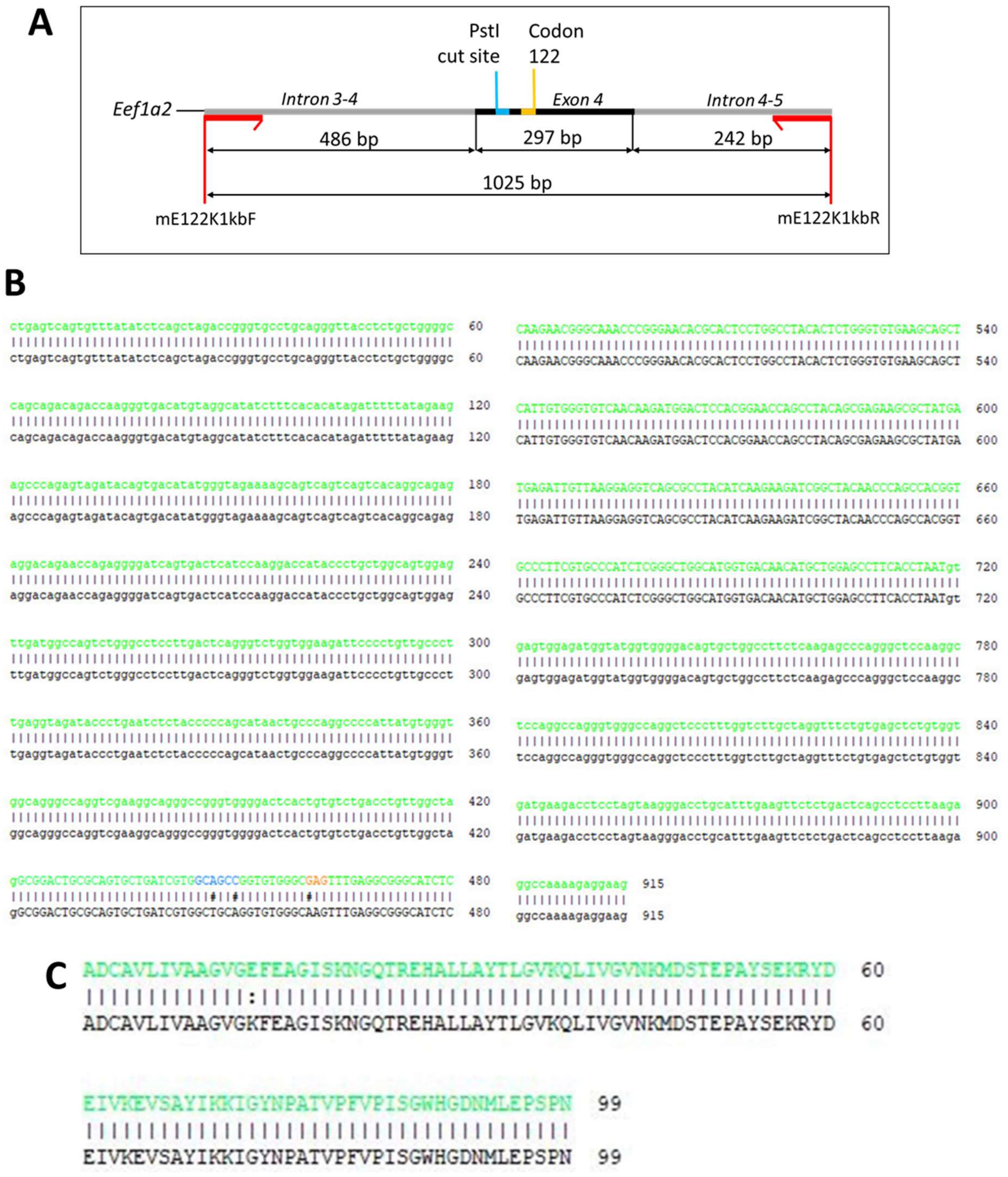

Fig. S3. On-target analysis of E122K CRISPR. A: Schematic of the 1025 bp region of genomic *Eef1a2* amplified by primers mE122K1KBF and mE122K1KBR. **B**: Alignment of wildtype *Eef1a2* sequence (green, assembly GRCm38.mm10) against *Eef1a2* sequence amplified from an E122K/E122K mouse (black) with primers mE122K1KBF and mE122K1KBR using a proofreading polymerase. Intronic sequence is in lowercase and exonic sequence is in uppercase. Codon 122 is shown in orange. The PstI restriction site codons are highlighted in blue. Mismatches are highlighted by hashes (#). **C**: Alignment of wildtype *Eef1a2* exon 4 protein sequence (green) against the protein sequence translated from the E122K/E122K DNA sequence shown in B. Mismatches are highlighted with a colon (:). B and C rendered in SerialCloner V2.6.1.

Disease Models & Mechanisms • Supplementary information

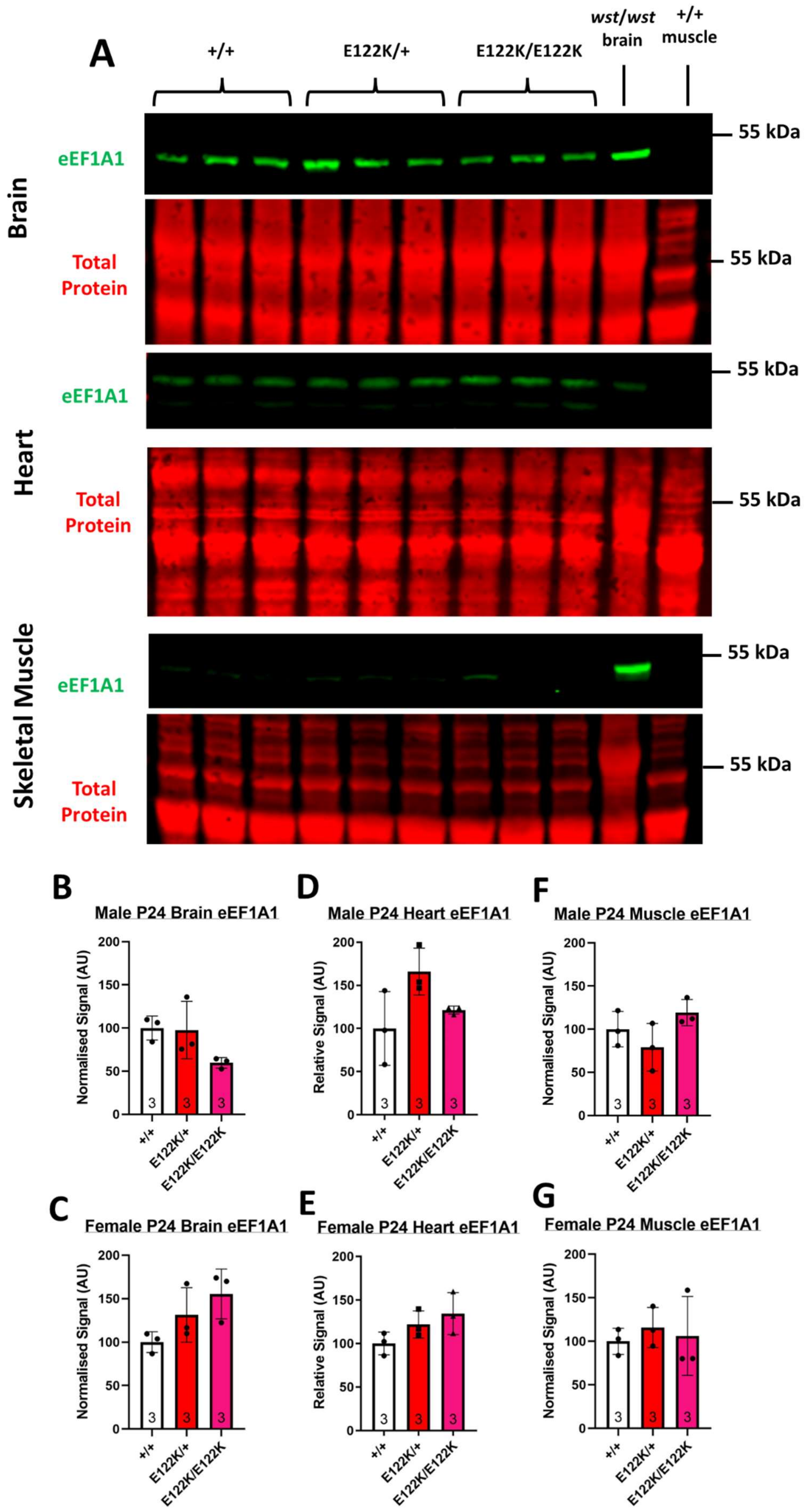

Fig. S4. eEF1A1 levels are not significantly altered in the brain, heart or skeletal muscle of E122K/+ or E122K/E122K mice at P24. A: representative Western blots of P24 brain, heart and muscle lysates showing eEF1A1 and a total protein stain. Brightness and contrast settings were set separately for each blot shown in panel A for illustration purposes (applied to the whole image), but this did not affect the quantification using Image Studio Lite software. **B-G**: quantified eEF1A1 levels, normalised to total protein and expressed as a percentage of wildtype, were compared using ordinary one-way ANOVAs. There were no statistically significant differences in relative eEF1A1 levels in male brain ($F(2, 6) = 3.46$, $p = 0.099$), female brain ($F(2, 6) = 3.576$, $p = 0.095$), male heart ($F(2, 6) = 3.929$, $p = 0.081$), female heart ($F(2, 6) = 2.726$, $p = 0.144$), male muscle ($F(2, 6) = 2.572$, $p = 0.156$) or female muscle ($F(2, 6) = 0.2058$, $p = 0.82$). Sample sizes are shown at the base of the bars. Error bars show the standard deviation.

Disease Models & Mechanisms • Supplementary information

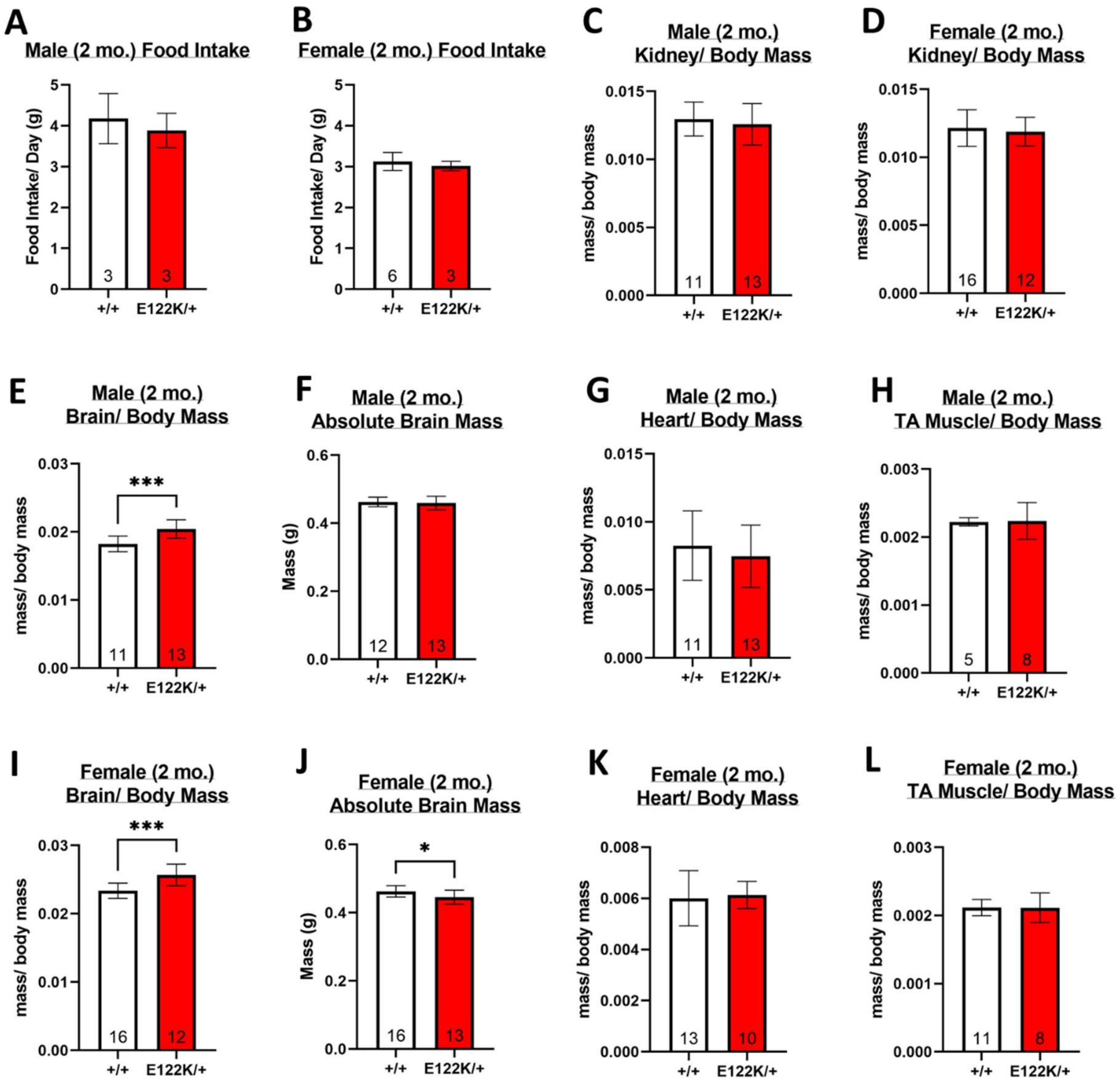

Fig. S5. analysis of body composition in E122K/+ mice. A & B: mean daily food intake in male (B) and female (C) mice at 2 months of age. The statistical units in A and B were single-sex, single-genotype cages containing 2-4 mice. **C-L**: mean mass of various organs/ tissues, given as either absolute mass (F & J) or as a fraction of total body mass. Genotypes in A, B, E-G, I, J and L were compared using unpaired t tests. Genotypes in H were compared using Welch's t test. Genotypes in C, D & K were compared using Mann-Whitney U tests. There was no statistically significant difference between genotypes for male food intake (t=0.6726, df=4, p = 0.5381), female food intake (t=0.8027, df=7, p = 0.4485), male kidney/ body ratio (U = 57, p = 0.4244), female kidney/ body ratio (U = 85, p = 0.6313), absolute male brain mass (t=0.4956, df=23, p = 0.6249), male heart/ body ratio (t=0.7861, df=22, p = 0.4402), female heart/ body ratio (U = 38, p = 0.1010), male *tibialis anterior*/ body ratio (Welch's t=0.1397, df=8.120, p = 0.8923) or female *tibialis anterior*/ body ratio (t=0.02035, df=17, p = 0.984). There were statistically significant differences between genotypes for male brain/ body ratio (t=4.258, df=22, p = 0.0003), female brain/ body ratio (t=4.502, df=26, p = 0.0001) and female absolute brain mass (t=2.466, df=27, p = 0.0203). * denotes p < 0.05 and *** denotes p < 0.001). Sample sizes are shown at the base of the bars. Error bars show the standard deviation.

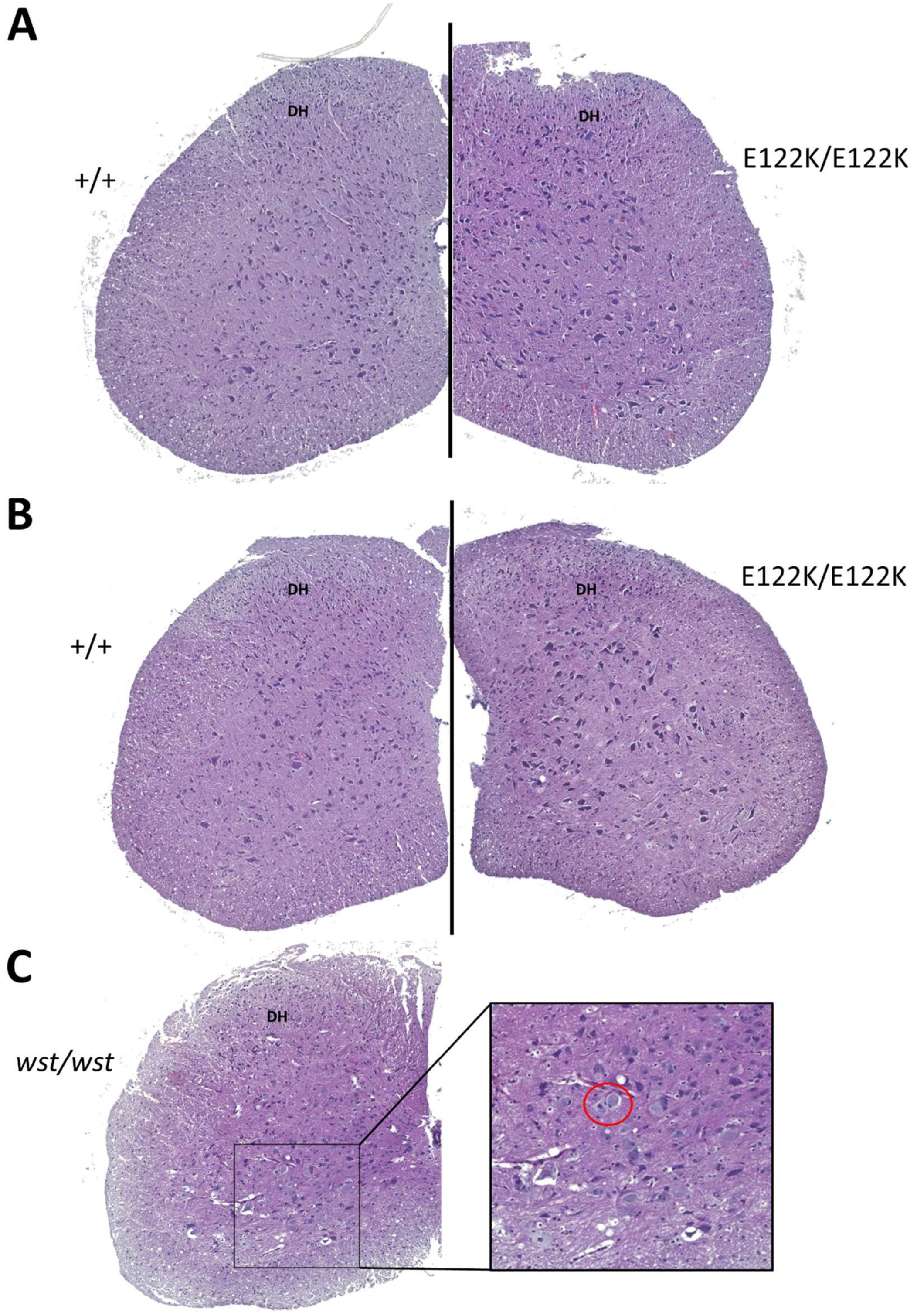

Fig. S6. E122K/E122K mice do not exhibit overt neurodegeneration. A & B: representative H&E stained 5 µm transverse sections through the cervical spinal cords of male (A) and female (B) +/+ and E122K/E122K mice. **C**: H&E stained 5 µm transverse sections through the cervical spinal cord of a P28 mouse homozygous for the *Eef1a2*-abolishing deletion *wst* (Shultz et al., 1982), showing conspicuous vacuolar neurodegeneration (circled). The dorsal horn (DH) is labelled in each section.

Disease Models & Mechanisms • Supplementary information

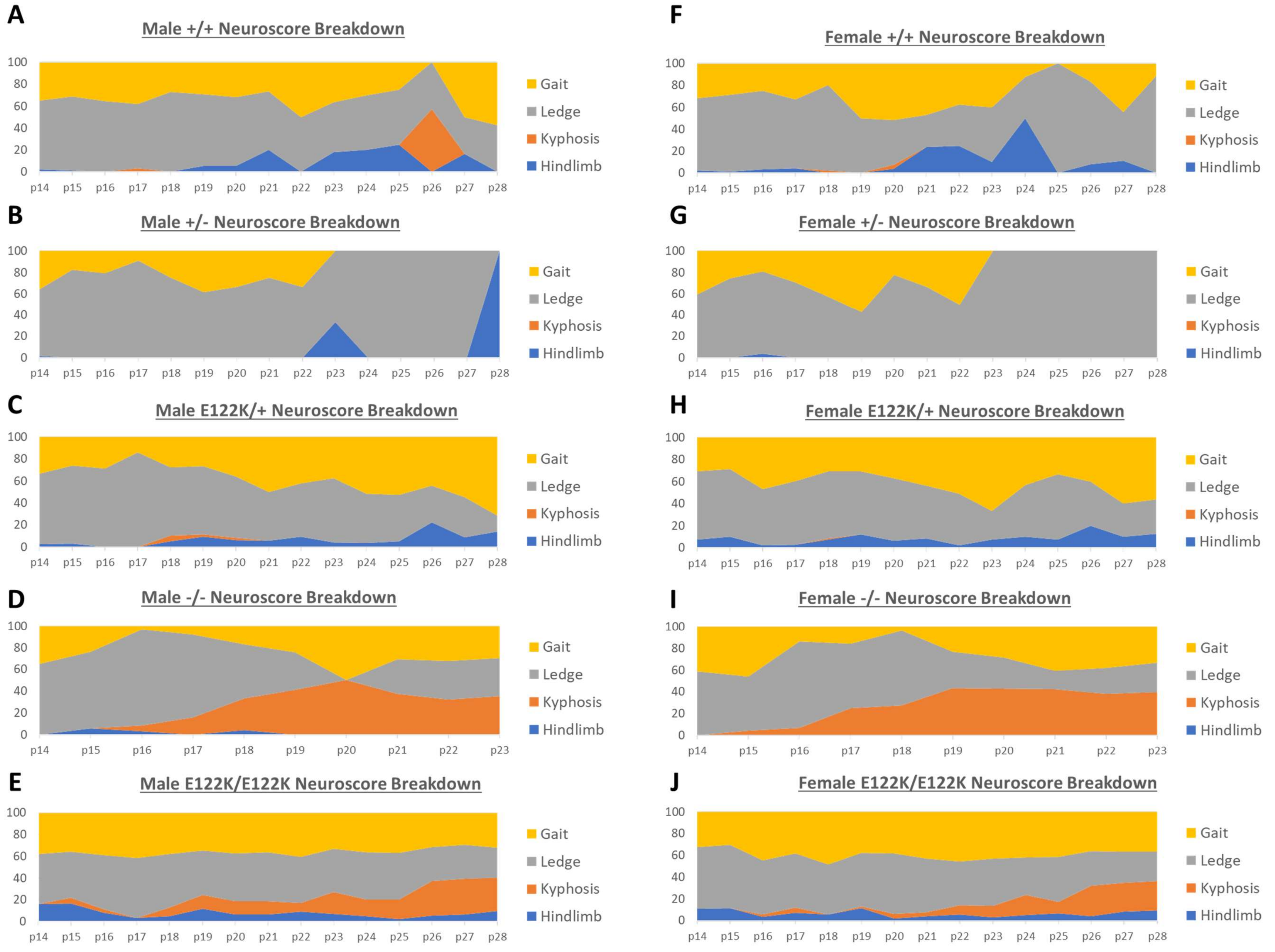

Fig. S7. breakdown of neuroscores. Contribution of each component to the total score at each given age in male (A-E) and female (F-J) mice in the E122K and Del22Ex3 lines between P14 and P28. These data were not statistically analysed.

Disease Models & Mechanisms • Supplementary information

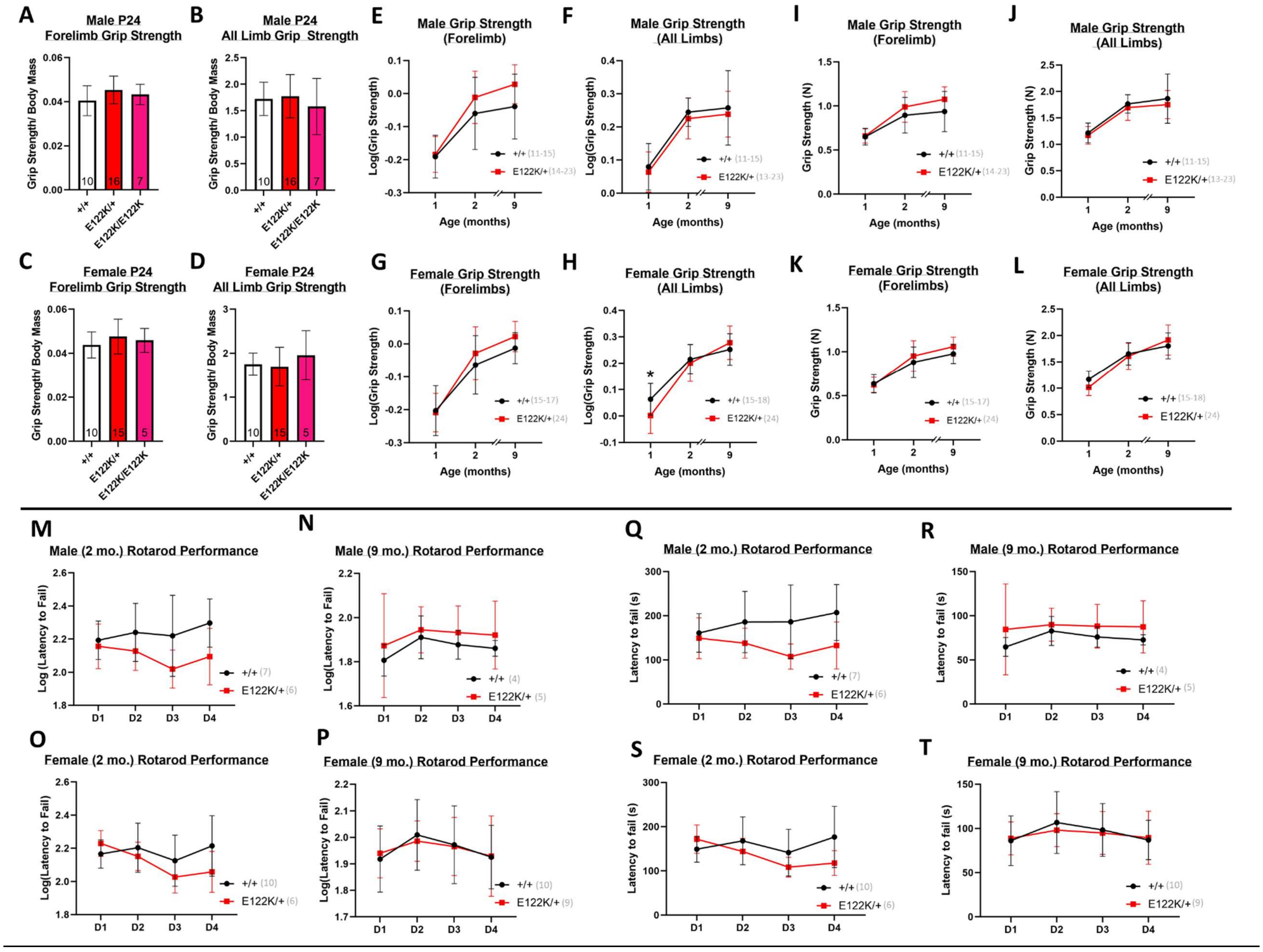

Disease Models & Mechanisms • Supplementary information

Fig. S8. mice carrying E122K do not show consistent grip strength deficits or progressive motor deficits. A-D: forelimb and all-limb grip strength in male and female mice at P24, normalised to total body mass. Genotypes in A-D were compared using ordinary one-way ANOVAs. There was no statistically significant difference between genotypes in A ($F(2, 30) = 1.919$, $p = 0.1644$), B ($F(2, 30) = 0.5512$, $p = 0.5820$), C ($F(2, 27) = 0.9031$, $p = 0.4172$), or D ($F(2, 27) = 0.7550$, $p = 0.4797$). **E-H**: absolute forelimb and all-limb grip strength in male and female mice between 1 and 9 months of age. As the body mass difference between +/+ and E122K/+ mice is not uniform over this age range, we compared absolute grip strengths in E-H. **I-L**: rotarod performance in male and female mice at 2 and 9 months of age, measured over four consecutive days at each age. Genotypes in E-H were compared using 2-way ANOVAs with an interaction term, factoring by genotype and age). Data in E-H were Log transformed in order to meet the assumptions of statistical tests (untransformed data are shown in I-L and were not statistically analysed). For male forelimb grip strength (E), there was no statistically significant interaction between genotype and age ($F(2, 85) = 1.282$, $p = 0.2829$) however there was a statistically significant effect of genotype ($F(1, 85) = 6.210$, $p = 0.0146$), with absolute grip strength in male E122K/+ mice being on average 10-15% higher than +/+ between 2-9 months of age. However, there were no statistically significant differences between genotypes at any age in Šídák's multiple comparisons test. These results are inconsistent with hypotonia and may reflect differences in anxiety and/ or motivation between genotypes in males. For male all-limb grip strength (F) there was no statistically significant effect of genotype ($F(1, 84) = 1.432$, $p = 0.2349$) or interaction ($F(2, 84) = 0.006694$, $p= 0.9933$). For female forelimb grip strength (G) there was no statistically significant effect of genotype ($F(1, 113) = 3.741$, $p = 0.0556$) or interaction ($F(2, 113) = 1.475$, $p = 0.2333$). For female all-limb grip strength (H), there was no statistically significant effect of genotype ($F(1, 114) = 1.985$, $p = 0.1616$) but there was a statistically significant interaction between genotype and age ($F(2, 114) = 4.535$, $p = 0.0127$). A significant difference between +/+ and E122K/+ all-limb grip strengths was detected at 1 month of age in Šídák's multiple comparisons test ($p = 0.0121$), with absolute all-limb grip strength being 13% lower than +/+ on average at 1 month of age. This difference was transient and likely reflects body mass differences to some degree. **M-P**: Rotarod performance at each age was compared between genotypes using 2-way ANOVAs (factoring by genotype and testing day), with repeated measures over testing days and the Geisser-Greenhouse correction applied. Data in M-P were Log transformed in order to meet the assumptions of statistical tests (untransformed data are shown in Q-T and were not statistically analysed). For males at 2 months (M), there was no statistically significant effect of genotype ($F(1, 11) = 3.914$, $p = 0.0735$) and no statistically significant interaction between genotype and testing day ($F(3, 33) = 1.573$, $p = 0.2143$). For females at 2 months (O), there was no statistically significant effect of genotype ($F(1, 14) = 1.260$, $p = 0.2805$). There was a statistically significant interaction between genotype and testing day ($F(3, 42) = 4.007$, $p = 0.0135$), but there were no statistically significant differences between genotypes on any specific testing day in Šídák's multiple comparisons test. For males at 9 months (N), there was no statistically significant effect of genotype ($F(1, 7)= 0.6477$, $p = 0.4474$) and no statistically significant interaction between genotype and testing day ($F(3, 21) = 0.04519$, $p = 0.9869$). For females at 9 months (P), there was no statistically significant effect of genotype ($F(1, 17) = 0.0005610$, $p = 0.9814$) and no statistically significant interaction between genotype and testing day ($F(3, 51) = 0.2518$, $p = 0.8597$). Sample sizes are shown at the base of the bars in A-D. Sample sizes are indicated in panel legends in E-T. All error bars show the standard deviation.

Disease Models & Mechanisms • Supplementary information

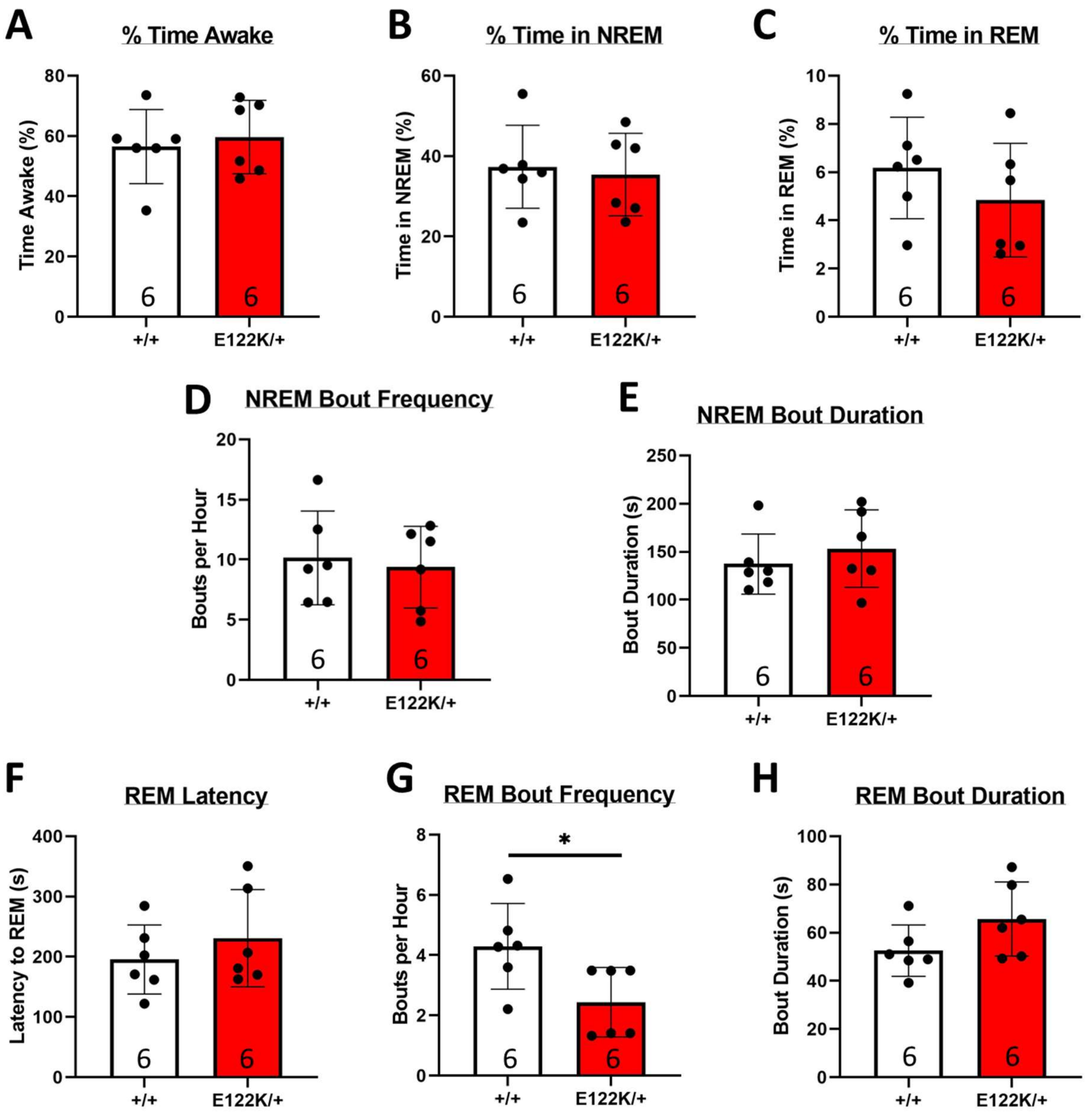

Fig. S9. E122K/+ mice exhibit normal sleep efficiency with possible alterations to REM architecture. A-C: mean percentage of recording spent awake (A), in NREM sleep (B) and in REM sleep (C). **D**: mean frequency of NREM sleep bouts. **E**: mean duration of NREM sleep bouts. **F**: mean latency to reach REM sleep during a sleep bout. **G**: mean frequency of REM bouts. **H**: mean duration of REM bouts. Genotypes in A-H were compared using unpaired t tests. * denotes $p < 0.05$. Sample sizes are shown at the base of the bars. Error bars show the standard deviation.

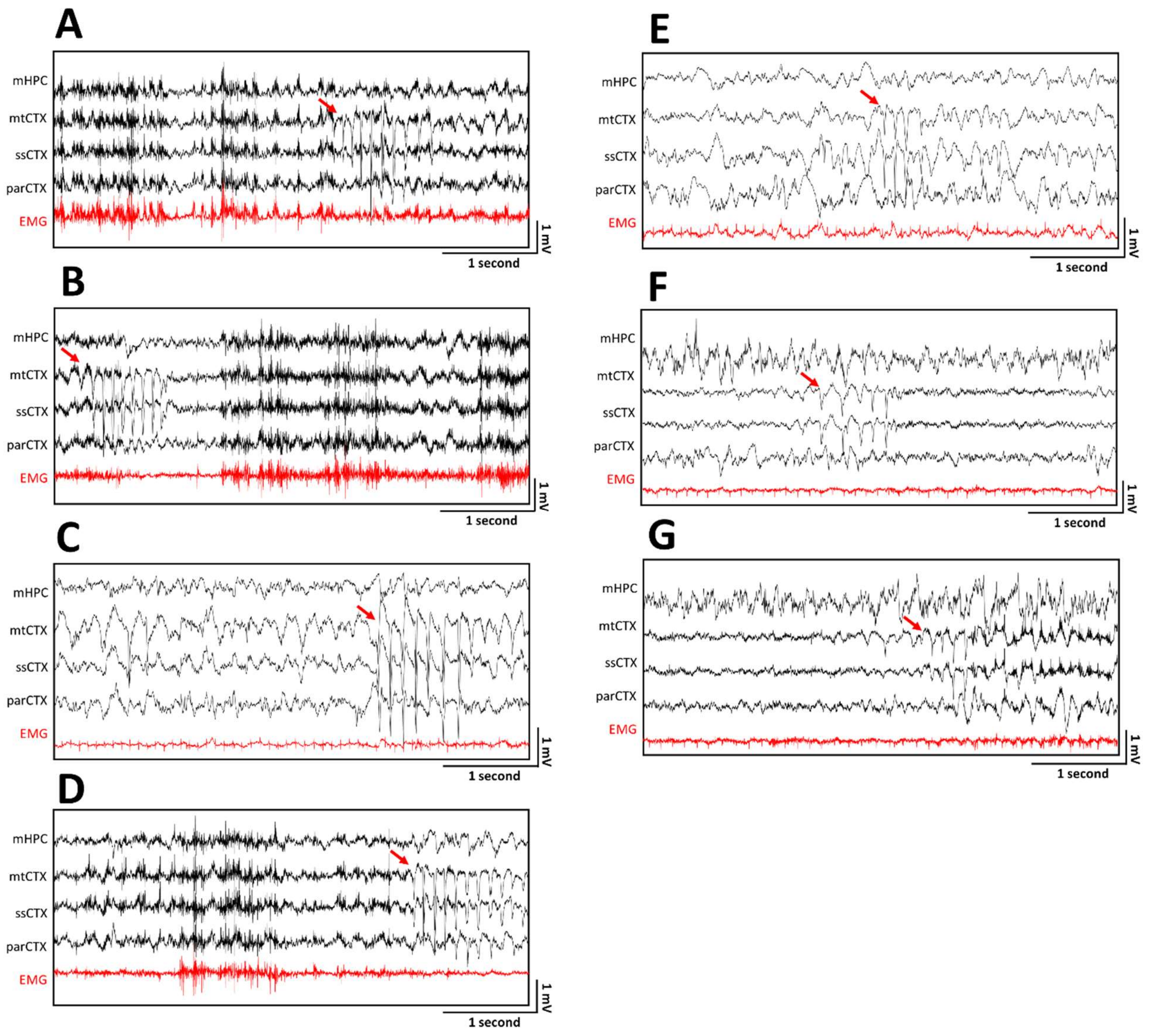

Fig. S10. Further CST examples. A-D: CSTs recorded in different vigilance states in E122K/+ mice. CSTs during waking were not consistently associated with EMG flattening (A vs. B). A CST occurring during the transition from waking to NREM is shown in panel D. **E-G:** three cortical polyspikes during sleep in +/+ mice. Event onsets are marked by red arrows.

Disease Models & Mechanisms • Supplementary information

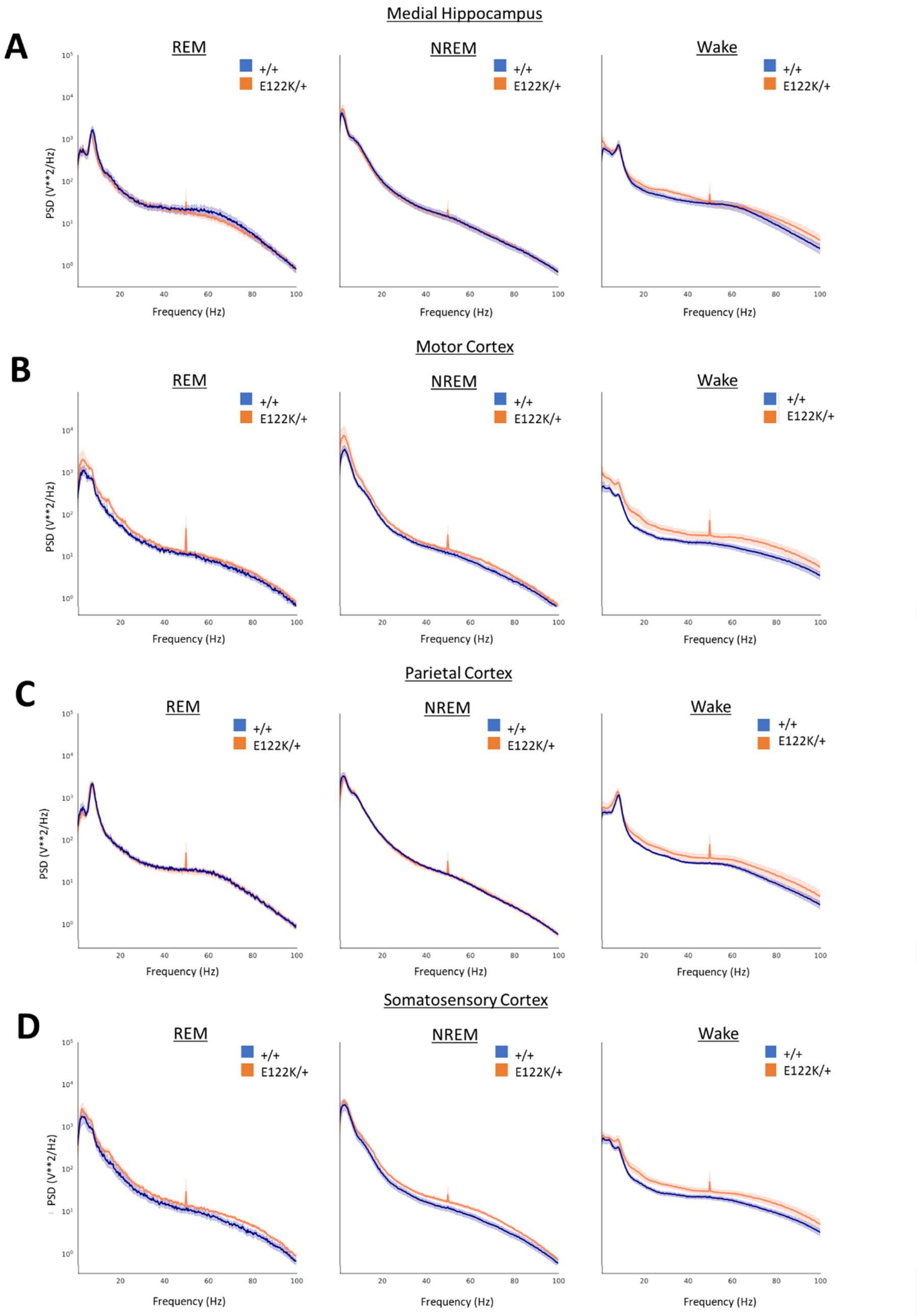

Fig. S11. Background EEG power spectra in +/+ and E122K/+ mice. A-D: power spectral density (PSD) plotted against frequency for each vigilance state in the medial hippocampus (A), motor cortex (B), parietal cortex (C) and somatosensory cortex (D). The shaded regions show the standard error at each frequency. The small peaks at 50 Hz are power line artefacts. These data were statistically analysed in Fig. S13.

Disease Models & Mechanisms • Supplementary information

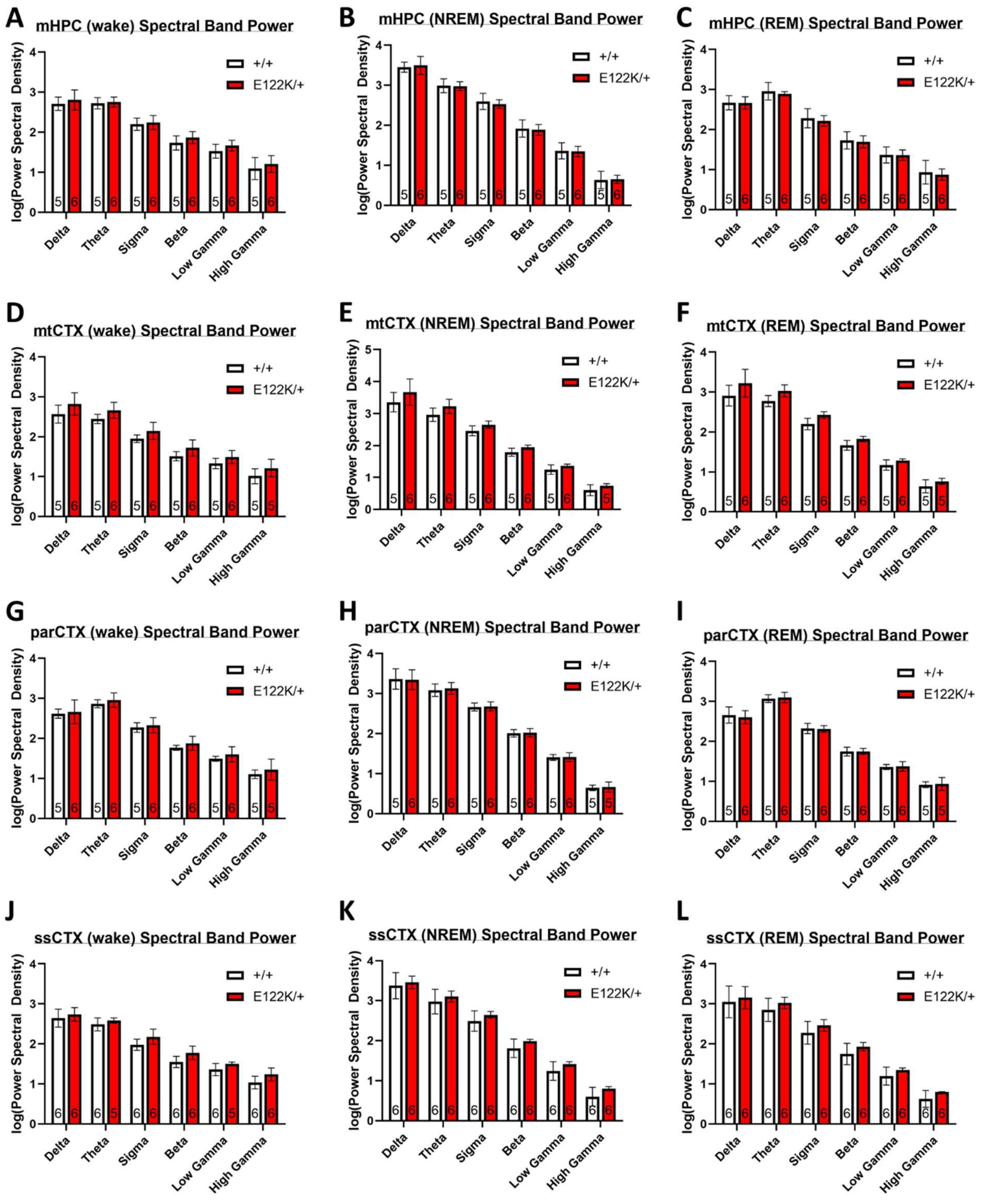

Fig. S12. Statistical comparison of EEG power spectra: mean PSDs for each animal binned into delta (1-4 Hz), theta (5-10 Hz), sigma (11-16 Hz), beta (16-30 Hz), low gamma (30-48 Hz) and high gamma (52-100 Hz) bands for each vigilance state in the medial hippocampus (mHPC, A-C), motor cortex (mtCTX, D-F), parietal cortex (parCTX, G-I) and somatosensory cortex (ssCTX, J-L). Data were log transformed in order to meet the assumptions of statistical tests and for illustration purposes. Genotypes in each recording location/ vigilance state were compared within spectral bands using unpaired t-tests (two-tailed) followed by correction for multiple comparisons (Holm-Šídák method). No statistically significant differences between genotypes were found. Sample sizes are shown at the base of the bars. Error bars show the standard deviation.

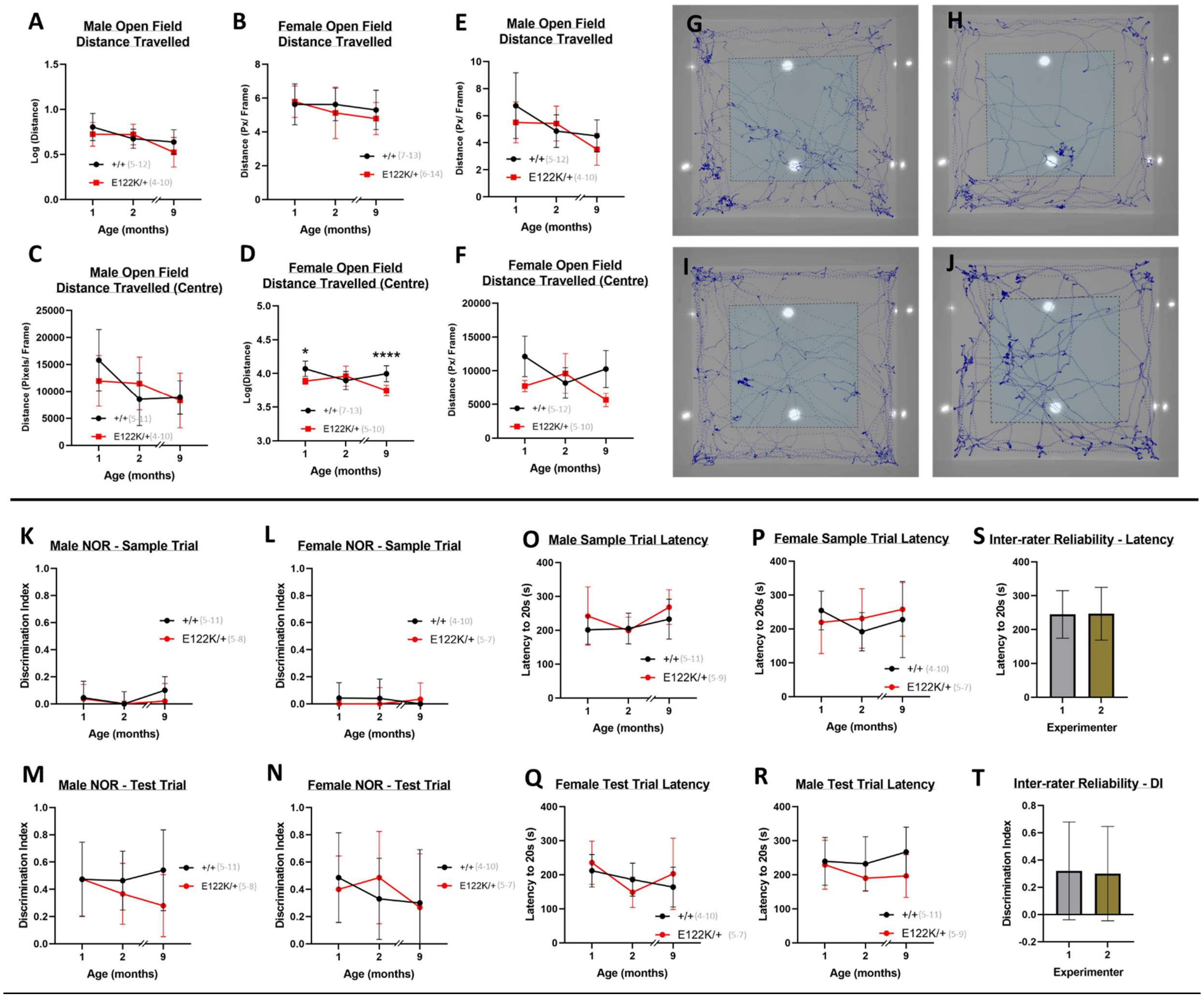

Disease Models & Mechanisms • Supplementary information

Fig. S13. Mice carrying E122K have normal locomotor activity levels and normal object recognition memory, with possible anxiety signatures. A & B: total distance travelled in the open field (expressed as pixels per video frame) for male (A) and female (B) mice. **C & D**: distance travelled in the open field centre zone for male (C) and female (D) mice. Data in A & D were Log transformed in order to meet the assumptions of statistical tests (untransformed data are shown in E & F and were not statistically analysed). Genotypes in A-D were compared using 2-way ANOVAs, factoring by genotype and age. For male open field distance (A), there was no statistically significant effect of genotype ($F(1, 46) = 1.582$, $p = 0.2148$) and no statistically significant interaction between genotype and age ($F(2, 46) = 1.827$, $p = 0.1724$). For male open field distance (B), there was no statistically significant effect of genotype ($F(1, 54) = 0.8522$, $p = 0.3600$) and no statistically significant interaction between genotype and age ($F(2, 54) = 0.4913$, $p = 0.6145$). For male centre distance, there was no statistically significant effect of genotype ($F(1, 45) = 0.1077$, $p = 0.7444$) and no statistically significant interaction between genotype and age ($F(2, 45) = 2.469$, $p = 0.0961$). For female centre distance, there was a statistically significant effect of genotype ($F(1, 49) = 14.45$, $p = 0.0004$) and no statistically significant interaction between genotype and age ($F(2, 49) = 8.956$, $p = 0.0005$). Šídák's multiple comparisons test revealed a statistically significant difference in female centre distance travelled at 1 month of age ($p = 0.0118$) and at 9 months of age ($p < 0.0001$), indicating increased thigmotactic behaviours. All animals at were naïve to the open field arena at 1 and 2 months, while the 9-month-old cohort consisted of a mixture of naïve and non-naïve animals. The effect of naivety on total distance travelled and distance travelled in the centre zone at 9 months of age was tested using 3-way ANOVA (factoring by genotype, naivety and sex), revealing no statistically significant first, second or third order effects of naivety on either open field measure (data not shown). G-J: Representative motion traces generated by EzTrack for open field recordings of a male wildtype mouse (G), a male E122K/+ mouse (H) a female wildtype mouse (I) and a female E122K/+ mouse (J) at 2 months of age. The 30x30 cm centre zone used for thigmotaxis measurements is annotated in blue. K-N: NOR object discrimination indices for the sample trials (K & L) and the test trials (M & N) for male and female mice between 1 and 9 months of age. O-R: latencies to reach 20 seconds of object interaction in the sample trials (O & P) and the test trials (Q & R) for male and female mice between 1 and 9 months of age. Data in K & L were not statistically analysed. Genotypes in M-R were compared using 2-way ANOVAs, factoring by genotype and age. For male test trial discrimination indices (M), there was no statistically significant effect of genotype ($F(1, 43) = 2.466$, $p = 0.1237$) and no statistically significant interaction between genotype and age ($F(2, 43) = 0.8965$, $p = 0.4155$). For female test trial discrimination indices (N), there was no statistically significant effect of genotype ($F(1, 33) = 0.01226$, $p = 0.9125$) and no statistically significant interaction between genotype and age ($F(2, 33) = 0.5193$, $p = 0.5997$). For male sample trial latencies (O), there was no statistically significant effect of genotype ($F(1, 43) = 2.031$, $p = 0.1613$) and no statistically significant interaction between genotype and age ($F(2, 43) = 1.004$, $p = 0.3747$). For female sample trial latencies (P), there was no statistically significant effect of genotype ($F(1, 33) = 0.1917$, $p = 0.6644$) and no statistically significant interaction between genotype and age ($F(2, 33) = 0.8536$, $p = 0.4351$). For male test trial latencies (O), there was no statistically significant effect of genotype ($F(1, 43) = 4.039$, $p = 0.0508$) and no statistically significant interaction between genotype and age ($F(2, 43) = 0.6844$, $p = 0.5098$). For female test trial latencies (P), there was no statistically significant effect of genotype ($F(1, 33) = 0.1670$, $p = 0.6854$) and no statistically significant interaction between genotype and age ($F(2, 33) = 1.411$, $p = 0.2582$). S & T: NOR tests were scored by two experimenters blind to genotype who trained on object interaction scoring together. To assess inter-rater reliability, ten NOR sessions were recorded and scored by each experimenter. S: mean latencies to reach 20 seconds of object interaction determined by each experimenter. T: mean discrimination indices calculated by each experimenter. Data in S & T were analysed using paired t-tests (two-tailed), with pairing based on the video recording. There was no statistically significant difference in the calculated discrimination indices ($p = 0.3434$) or interaction latencies ($p = 0.8393$) between experimenters. Sample sizes are indicated in panel legends. All error bars show the standard deviation.

Disease Models & Mechanisms • Supplementary information

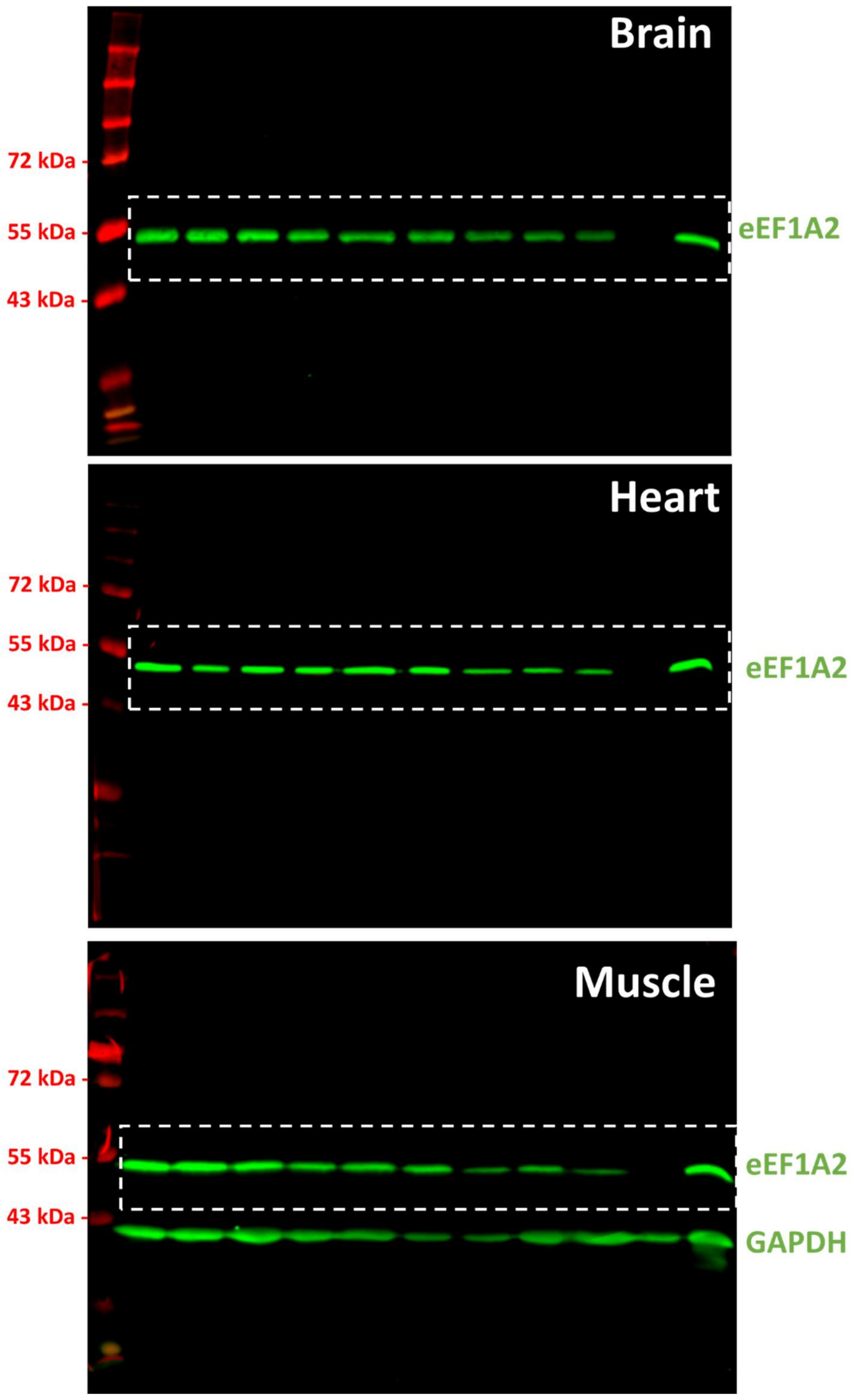

Fig. S14. Blot transparency. Uncropped fluorescent immunoblots for the eEF1A2 Westerns shown in Fig. 1C. The cropped region is highlighted. The muscle blot was additionally probed for GAPDH. Protein ladder is Colour Prestained Protein Standard (New England Biolabs #P7719S).

Disease Models & Mechanisms • Supplementary information

Table S1. Summary of clinical findings in individuals heterozygous for mutation E122K

Sex	Epilepsy?	Intellectual Disability/ Developmental Delay?	Autism?	Other features	Reference
M	Myoclonic absences (onset at 4y). Controlled at 4y followed by relapse at 7y and partial remission by 13y.<br><br>Abnormal EEG (intermittent delta/theta activity at 4y. Focal spikes/ waves at 13y).	Severe ID, developmental regression after seizure onset at 7y	ASD	Non-verbal, delayed psychomotor development. Walked unaided by 24mo, motor incoordination & ataxic gait at 13y.<br><br>Periventricular hyperintensities on MRI at 1.6y (non-progressive). Failure to thrive. Abnormal facial features. Sleep disorder requiring medication.	(De Rinaldis et al., 2020)
M	Myoclonic & myoclonic-atonic seizures (onset at 8mo), followed by atypical absences. Lennox-Gastaut syndrome from 5y. Initially treatment resistant but later partially controlled.<br><br>Abnormal EEG (diffuse polyspikes and multifocal spikes during sleep, continuous parietal delta activity during waking).	Severe ID, developmental arrest following emergence of delta activity.	NR	Abnormal facial features, hypotonia, unable to stand unaided or speak meaningful words. Cerebral atrophy at 1 year 9 months on MRI.	(Inui et al., 2016, 2020)
F	Myoclonic & atypical absence seizures (onset at 10mo). Lennox-Gastaut syndrome from 5 years. Initially treatment resistant but later partially controlled.	Severe ID, developmental arrest following emergence of delta activity.	NR	Abnormal facial features, cannot speak meaningful words, hypotonia, unable to hold up head or roll over, progressive cerebral atrophy on MRI.	(Inui et al., 2016, 2020)

Disease Models & Mechanisms • Supplementary information

	Abnormal EEG (diffuse spikes, waves and multifocal spikes during sleep, continuous parietal delta activity during waking).				
F	Infantile/ nodding spasms (onset at 4mo). Controlled at 12y.<br><br>Abnormal EEG (multifocal spikes, polyspikes and spike-wave discharges)	Severe ID	Autistic features, self-injurious behaviours	Neonatal hypotonia. Delayed psychomotor development (walked independently from 6 years 10 months), ataxic gait (at 12 years), abnormal facial features, sleep disorder requiring medication (from 1 year), speaks no meaningful words. Progressive cerebral atrophy on MRI by 3 years. Failure to thrive, talipes varus	(Nakajima et al., 2015)
F	Infantile spasms (onset at 10wks), followed by head nodding. Controlled.<br><br>Abnormal EEG (spikes, polyspikes and slow wave activity at 21 mo).	Severe ID	NR	Neonatal hypotonia, delayed psychomotor development (walked independently by 4 years but unsteady gait), non-verbal (uses signs), abnormal facial features, microcephaly.	(Lam et al., 2016)
M	Focal seizures (onset at 3mo), followed by reflex myoclonic (startle) seizures and infantile spasms. Controlled at 25 years.<br><br>Abnormal EEG (frequent generalised paroxysms at 9 months; symmetric slowing without discharges at 14 years).	Severe ID	Self-injurious behaviour.	Speaks in single words. Ambulatory but ataxic. Hypotonia, strabismus, frequent childhood infections, abnormal facial features. Acquired microcephaly; MRI normal at 32 years.	(Carvill et al., 2020)
M	Myoclonic seizures (onset at 4mo), followed by tonic, myoclonic atonic, tonic-clonic and absence seizures. Treatment resistant.	Moderate-severe ID	Autistic features	Non-verbal, non-ambulatory by 3y, hypotonia. MRI normal at 2 years.	(Carvill et al., 2020)

Disease Models & Mechanisms • Supplementary information

	Abnormal EEG (multifocal spiking, background delta/theta activity).				
F	Myoclonic seizures (onset at 2mo), followed by tonic and atypical absence seizures. Controlled.<br><br>Abnormal EEG (infrequent generalised polyspikes and spike-waves).	NR	NR	Non-verbal, non-ambulatory as of 2y, hypotonia, hyperreflexia, choreoathetosis, cortical visual impairment. Left parieto-occipital T2 hyperintensities on MRI.	(Carvill et al., 2020)
M	Early infantile epileptic encephalopathy (onset at 3mo).	NR	NR	NR	(Lee et al., 2021)

NR = not reported. ID = intellectual disability. MRI = magnetic resonance imaging. Two further patients heterozygous for E122K have been described in the DECIPHER database but are not shown (Firth et al., 2009).

Table S2. Primers used

Primer Name	Sequence (5' – 3')	Target	Expected Amplicon Size (bp)
mE122KTOPOF	TGGAAGATTCCCCTGTTGCC	*Eef1a2*	432
mE122TOPOR	AAGGCTCCAGCATGTTGTCA		
mE122KGenoF	GAAGATTCCCCTGTTGCCCT	*Eef1a2*	460
mE122KGenoR	CCACCATACCATCTCCACTCA		
mE122K1kbF	GCAGACCTCTACCCATGT	*Eef1a2*	1025
mE122K1kbR	GGGATGACTTGATGAAACC		
OT1F	CTTCTGTGGAGGATGGACCAT	CRISPR off-target locus 1	316
OT1R	TGACTCTCTATTAGAACCCCTGGC		
OT2F	CCTTACCACGTTGGGAAACAT	CRISPR off-target locus 2	306
OT2R	TTACTGCAAAAGTCCATCTGCC		
OT3F	CTGTCCAAGAGCAGATGAATACG	CRISPR off-target locus 3	295
OT3R	AGCTGAGCACTGAGAGCACC		
OT4F	CCTGCCATCTGTCCATTTTG	CRISPR off-target locus 4	400
OT4R	CCTCAGAACTCCCATGGACTAAGT		
OT5F	GATGGGAGGAACGGTACAAGTC	CRISPR off-target locus 5	256
OT5R	AGGCACTCCTCCATGGACA		
OT6F	GGATCTGAAGGATGTGCTGC	CRISPR off-target locus 6	345
OT6R	CCCTCATAGCAGATAAGGCCTTC		
OT7F	CTGATCTCAGGTGCTCTGTAA	CRISPR off-target locus 7	414
OT7R	CCCACAGGAGGAAAACCAA		
OT8F	CCATGGTTTATCACAGCAAGGA	CRISPR off-target locus 8	319
OT8R	GGCTCGTCACCGTTAGCAT		

Disease Models & Mechanisms • Supplementary information

OT9F	GTCATTCTCAAAAGAAGCATGGG	CRISPR off-target locus 9	367
OT9R	CCGGCTGGAAACTCTCTATCC		
OT10F	AAACCCACCATCCCATCCC	CRISPR off-target locus 10	336
OT10R	CCCAATGAAGGAGAAAGCAC		
m1A2qPCRF	GCCACGATCAGCACTGCG	*Eef1a2* mRNA	246
m1A2qPCRR	CAAGCGGACCATCGAGAAGT		
m1A1qPCRF	ACGAGGCAATGTTGCTGGTGAC	*Eef1a1* mRNA	132
m1A1qPCRR	GTGTGACAATCCAGAACAGGAGC		
mGAPDHqPCRF	GGAAGGGCTCATGACCACA	*Gapdh* mRNA	165
mGAPDHqPCRR	CCGTTCAGCTCTGGGATGAC		
mUBCqPCRF	AGCCCAGTGTTACCACCAAG	*Ubc* mRNA	97
mUBCqPCRR	ACCCAAGAACAAGCACAAGG		
mB2MqPCRF	ATTCACCCCCACTGAGACTG	*B2m* mRNA	193
mB2MqPCRR	TGCTATTTCTTTCTGCGTGC		

Disease Models & Mechanisms • Supplementary information

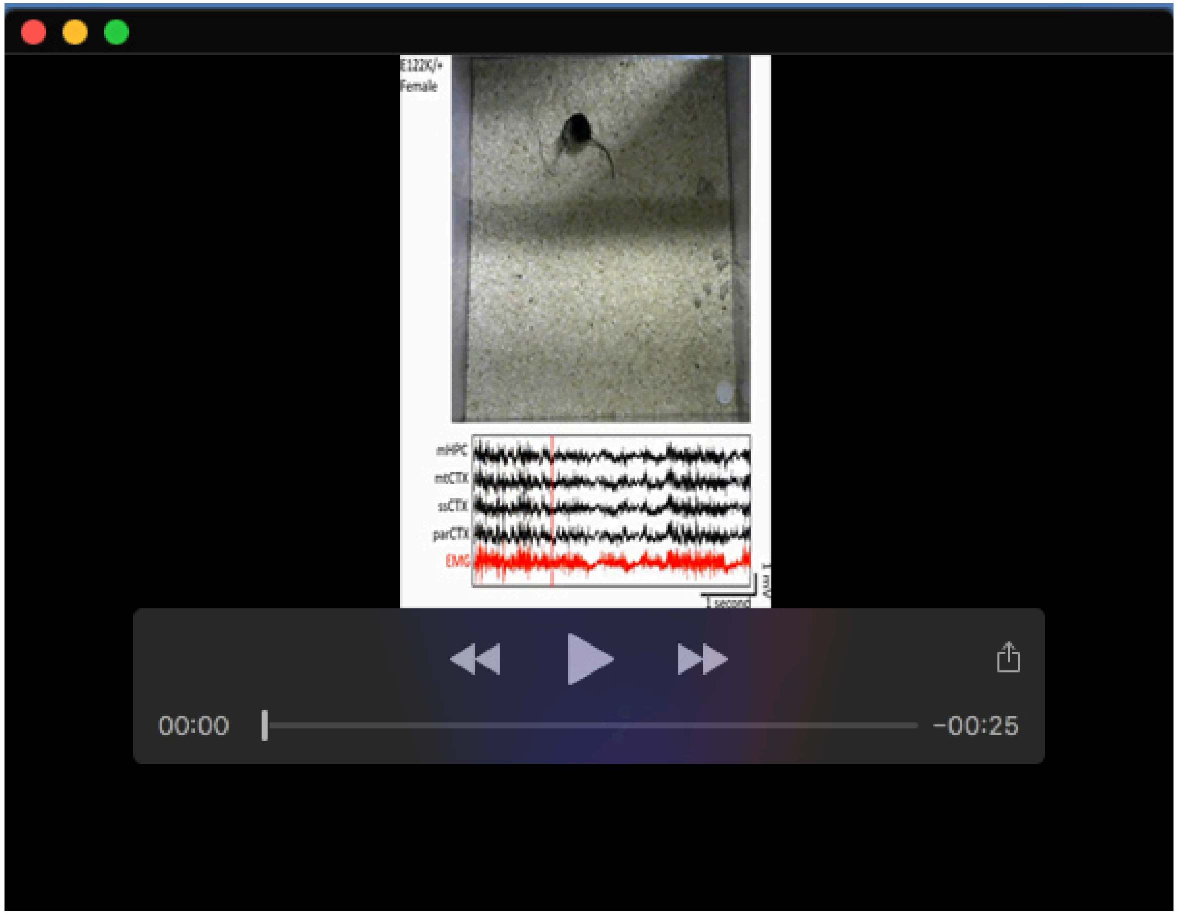

Movie 1. Electrographic seizures in E122K/+ mice have no behavioural correlates. Video shows a selection of electrographic seizures occurring in E122K/+ mice during waking. EEG/EMG montage is shown for each clip.

Supplementary references

De Rinaldis, M., Giorda, R. and Trabacca, A. (2020). Mild epileptic phenotype associates with de novo eef1a2 mutation: case report and review. *Brain Dev.* **42**, 77-82. doi:10.1016/j.braindev.2019.08.001

Firth, H. V., Richards, S. M., Bevan, A. P., Clayton, S., Corpas, M., Rajan, D., Van Vooren, S., Moreau, Y., Pettett, R. M. and Carter, N. P. (2009). DECIPHER: database of chromosomal imbalance and phenotype in humans using ensembl resources. *Am. J. Hum. Genet.* **84**, 524-533. doi:10.1016/j.ajhg.2009.03.010

Lee, H.-F., Chi, C.-S. and Tsai, C.-R. (2021). Diagnostic yield and treatment impact of whole-genome sequencing in paediatric neurological disorders. *Dev. Med. Child Neurol.* **63**, 934-938. doi:10.1111/dmcn.14722
